# Downregulation of extramitochondrial BCKDH and its uncoupling from AMP deaminase in type 2 diabetic OLETF rat hearts

**DOI:** 10.14814/phy2.15608

**Published:** 2023-02-17

**Authors:** Toshifumi Ogawa, Hidemichi Kouzu, Arata Osanami, Yuki Tatekoshi, Tatsuya Sato, Atsushi Kuno, Yugo Fujita, Shoya Ino, Masaki Shimizu, Yuki Toda, Wataru Ohwada, Toshiyuki Yano, Masaya Tanno, Takayuki Miki, Tetsuji Miura

**Affiliations:** ^1^ Department of Cardiovascular, Renal and Metabolic Medicine Sapporo Medical University School of Medicine Sapporo Japan; ^2^ Department of Cellular Physiology and Signal Transduction Sapporo Medical University School of Medicine Sapporo Japan; ^3^ Department of Pharmacology Sapporo Medical University School of Medicine Sapporo Japan; ^4^ Department of Clinical Pharmacology, Faculty of Pharmaceutical Sciences Hokkaido University of Science Sapporo Japan

**Keywords:** AMP deaminase, branched‐chain amino acids, branched‐chain α‐keto acid dehydrogenase, diabetic cardiomyopathy

## Abstract

Systemic branched‐chain amino acid (BCAA) metabolism is dysregulated in cardiometabolic diseases. We previously demonstrated that upregulated AMP deaminase 3 (AMPD3) impairs cardiac energetics in a rat model of obese type 2 diabetes, Otsuka Long‐Evans‐Tokushima fatty (OLETF). Here, we hypothesized that the cardiac BCAA levels and the activity of branched‐chain α‐keto acid dehydrogenase (BCKDH), a rate‐limiting enzyme in BCAA metabolism, are altered by type 2 diabetes (T2DM), and that upregulated AMPD3 expression is involved in the alteration. Performing proteomic analysis combined with immunoblotting, we discovered that BCKDH localizes not only to mitochondria but also to the endoplasmic reticulum (ER), where it interacts with AMPD3. Knocking down AMPD3 in neonatal rat cardiomyocytes (NRCMs) increased BCKDH activity, suggesting that AMPD3 negatively regulates BCKDH. Compared with control rats (Long‐Evans Tokushima Otsuka [LETO] rats), OLETF rats exhibited 49% higher cardiac BCAA levels and 49% lower BCKDH activity. In the cardiac ER of the OLETF rats, BCKDH‐E1α subunit expression was downregulated, while AMPD3 expression was upregulated, resulting in an 80% lower AMPD3‐E1α interaction compared to LETO rats. Knocking down E1α expression in NRCMs upregulated AMPD3 expression and recapitulated the imbalanced AMPD3‐BCKDH expressions observed in OLETF rat hearts. E1α knockdown in NRCMs inhibited glucose oxidation in response to insulin, palmitate oxidation, and lipid droplet biogenesis under oleate loading. Collectively, these data revealed previously unrecognized extramitochondrial localization of BCKDH in the heart and its reciprocal regulation with AMPD3 and imbalanced AMPD3‐BCKDH interactions in OLETF. Downregulation of BCKDH in cardiomyocytes induced profound metabolic changes that are observed in OLETF hearts, providing insight into mechanisms contributing to the development of diabetic cardiomyopathy.

## Graphical abstract

The present study revealed previously unrecognized extramitochondrial localization of branched‐chain α‐keto acid dehydrogenase (BCKDH) and its reciprocal regulation with AMPD3 in cardiomyocytes and imbalanced AMPD3‐BCKDH interactions in a rat model of type 2 diabetes, Otsuka Long‐Evans‐Tokushima fatty (OLETF). Downregulation of BCKDH in cardiomyocytes induced profound metabolic changes that are observed in OLETF hearts, providing insight into mechanisms contributing to the development of diabetic cardiomyopathy.

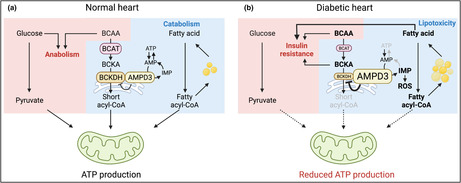

## 1 INTRODUCTION

Diabetic cardiomyopathy is defined as cardiac dysfunction involving structural, functional, and metabolic changes not associated with coronary artery disease (Miki et al., 2013). Its metabolic hallmark is reduced substrate flexibility, with augmented fatty acid uptake and oxidation and suppressed glucose oxidation (Miki et al., 2013). The augmented influx of fatty acids leads to intracellular accumulation of various lipid species and is believed to cause cardiac lipotoxicity (Lopaschuk et al., 2010; Wende & Abel, 2010). However, increasing evidence suggests that not all the accumulated lipid species are necessarily harmful (Goldberg et al., 2018). Specifically, triglyceride‐containing lipid droplet biogenesis in the endoplasmic reticulum (ER) reportedly sequesters potentially toxic fatty acids in excess (Listenberger et al., 2003; Liu et al., 2009). Lipid droplets serve not only as efficient reservoirs of fatty acids for mitochondrial oxidation (Banke et al., 2010) but also as essential sources of lipid ligands for peroxisome proliferator‐activated receptor‐α (PPARα) activation, a critical step in fatty acid metabolism (Haemmerle et al., 2011). A more detailed understanding of diabetes‐induced metabolic remodeling may lead to metabolic therapies for diabetic cardiomyopathy.

Myocardial fatty acid metabolism is tightly coupled with amino acid catabolism in normal hearts (Murashige et al., 2020). When fatty acids are dominant fuel sources, such as in fasting, the heart takes up branched‐chain amino acids (BCAAs) and glutamate. In addition, the heart secretes nitrogen‐rich amino acids, such as glutamine. Although the contribution of BCAA oxidation to mitochondrial ATP production is negligible (Fillmore et al., 2018; Murashige et al., 2020; Neinast, Jang, et al., 2019), experimental disruption of BCAA catabolism consistently induced maladaptive cardiac remodeling (Li et al., 2017; Lu et al., 2007; Sun et al., 2016), suggesting that BCAAs play crucial roles in addition to being energy substrates. Given the emerging evidence showing that systemic BCAA metabolism is dysregulated in cardiometabolic disease (Lynch & Adams, 2014; White & Newgard, 2019), it is tempting to speculate that impaired myocardial BCAA metabolism is somehow involved in the metabolic remodeling of diabetic cardiomyopathy and maybe a potential therapeutic target.

We recently reported that upregulated expression of AMP deaminase 3 (AMPD3), a rate‐limiting enzyme in the purine nucleotide cycle, is involved in abnormal cardiac energetics in Otsuka Long‐Evans‐Tokushima fatty (OLETF) rats, models of type 2 diabetes (T2DM) (Igaki et al., 2021; Kouzu et al., 2015; Tatekoshi et al., 2018). The elevated AMPD activity was associated with ATP depletion during acute pressure overload due to excessive degradation of adenine nucleotides (Kouzu et al., 2015; Tatekoshi et al., 2018), which subsequently facilitated reactive oxygen species production via downstream xanthine oxidoreductase and led to mitochondrial respiratory failure (Igaki et al., 2021). Meanwhile, AMPD3 was found to play a central role in coupling fatty acid and BCAA metabolism in skeletal muscle (Hong et al., 2017). However, the roles of AMPD3 in cardiac BCAA metabolism and their modifications by T2DM have not been examined.

Considering these findings, we hypothesized that the cardiac BCAA levels and the activity of branched‐chain α‐keto acid dehydrogenase (BCKDH), a rate‐limiting enzyme complex in BCAA catabolism that is localized in the mitochondrial matrix, are altered by T2DM, and that upregulated AMPD3 expression is involved in the alteration. To test this hypothesis, we used OLETF rats as models of T2DM since they are suitable for use in elucidating the role of impaired BCAA metabolism in T2DM. OLETF rats have higher circulating BCAAs and lower hepatic BCKDH activity (Kuzuya et al., 2008); therefore, these rats recapitulate dysregulation of BCAA metabolism in patients with T2DM (Lynch & Adams, 2014).

## 2 MATERIALS AND METHODS

### 2.1 Animal models

Animal studies were conducted according to the Guide for the Care and Use of Laboratory Animals published by the National Research Council of the National Academies, USA (2011) and was approved by the Animal Use Committee of Sapporo Medical University. In this study, we primarily used male OLETF rats and Long‐Evans Tokushima Otsuka (LETO) rats that were 34–40 weeks old. OLETF develops T2DM by hyperphagia. In our previous studies, we repetitively confirmed elevation of body weight, plasma glucose, serum triglycerides, and plasma insulin levels in OLETF at the age of 29–35 weeks compared with those in LETO (Hotta et al., 2010; Kouzu et al., 2015; Miki et al., 2009; Tatekoshi et al., 2018), and insulin resistance in OLETF at the same ages was demonstrated by the glucose clamp method (Hotta et al., 2010). We also used 9–10‐week‐old Sprague–Dawley rats to examine the intracellular localization of AMPD3 and BCKDH. Rats were purchased from Sankyo Labo Service Corporation. The rats were housed in standard plastic cages with paper chip bedding and maintained in a 14 h/10 h light–dark cycle temperature‐controlled room (22 ± 1°C) with free access to water and a standard rodent chow (CRF‐1, Charales River Laboratories). Data for myocardial BCAA levels were retrieved from metabolomics database obtained in our previous study (Kouzu et al., 2015).

### 2.2 Cell culture

H9c2 and HEK293 cells (American Type Culture Collection) were cultured in DMEM (Wako 044‐29765) or DMEM/F‐12 (Gibco 11320‐033) supplemented with 10% fetal bovine serum (FBS) at 37°C with 5% CO_2_. The cells were used for experiments when they were 70%–90% confluent. Neonatal rat cardiomyocytes (NRCMs) were isolated as previously described (Tatekoshi et al., 2018). NRCMs were plated on fibronectin‐coated plates in culture medium; 500 mL of Medium 199 (Gibco 11150‐059) was fortified by the addition of 5 mL of MEM (Gibco 11140‐050), 5 mL l‐glutamine (200 mM, Gibco 25030‐081), 1.75 g of glucose, 1 mg of vitamin B12, 5 mL of HEPES (1 M, Gibco 15630‐080), 10% FBS and 100 μM bromodeoxyuridine. The final glucose concentration was 25 mM. The culture medium was replaced with fresh medium containing 2% FBS the next day.

### 2.3 Transfection

siRNAs were purchased from Dharmacon. The following siRNAs were used in this study: rat AMPD3 (M‐091904‐01), rat BCKDHA (M‐081866‐01), and non‐targeting control (D‐001206‐13, D‐001810‐10). The siRNAs were transfected using Dharmafect 1 transfection reagent (Dharmacon T‐2001) or RNAiMAX (Thermo Fisher Scientific 13778). Experiments were performed 24–48 h after transfection. FLAG‐AMPD3 or FLAG‐control vector was transfected into H9c2 cells using FuGENE HD (Promega E2311) as previously described (Tatekoshi et al., 2018).

### 2.4 BCKDH activity assay

Tissue BCKDH activity was measured as previously described with slight modification (White et al., 2018). Briefly, 50 μL of tissue homogenate was added to 300 μL of assay buffer (50 mM HEPES, pH 7.5; 30 mM Kpi pH 7.5; 0.4 mM CoA; 3 mM NAD^+^; 5% FBS; 2 mM thiamine pyrophosphate; 2 mM MgCl_2_; and 86.6 μM α‐keto [1‐^14^C] isovalerate, 3838 cpm/nmol of specific radioactivity) in a 1.7 mL Eppendorf tube, which was immediately connected to another tube containing a raised 1 M NaOH CO_2_ trap. The tubes were sealed with parafilm and incubated for 30 min at 37°C. The reaction mixture was acidified by the injection of 70% perchloric acid and reconnected to the CO_2_ trap tube, where it remained at room temperature for 1 h. The ^14^CO_2_ contained in the trap was counted in a liquid scintillation counter (AccuFLEX LSC‐8000). An assay with intact cells was conducted as previously described (Chuang & Chuang, 2000).

### 2.5 Measurement of cardiac AMP deaminase activity

AMP deaminase activity was measured as previously described (Igaki et al., 2021; Kouzu et al., 2015; Tatekoshi et al., 2018).

### 2.6 Tissue and cell fractionation

Fractionation of tissues was performed as previously reported (Ma et al., 2017; Wieckowski et al., 2009) with slight modification. Briefly, 0.5 g of freshly harvested tissue was manually homogenized with a Dounce homogenizer in 4 mL of ice‐cold buffer (225 mM mannitol, 75 mM sucrose, 0.5% BSA, 30 mM Tris–HCl (pH 7.4), and 0.5 mM EGTA). Nuclei and unbroken cells were pelleted by centrifugation. The supernatant was collected and centrifuged to separate crude mitochondria from the cytosol and ER fractions. After two washes, the crude mitochondrial fraction was suspended in 0.5 mL of mitochondrial resuspension buffer (MRB, 250 mM mannitol, 5 mM HEPES (pH 7.40), and 0.5 mM EGTA), layered on 4 mL of Percoll medium (225 mM mannitol, 25 mM HEPES (pH 7.4), 1 mM EGTA, and 30% Percoll (vol/vol)) and less than 0.5 mL of MRB, and then centrifuged at 95,000 *g* for 30 min. The pure mitochondrial fraction was collected from the Percoll gradient and centrifuged to obtain a pellet. The supernatant containing the cytosol and ER fractions was centrifuged at 20,000 *g* for 60 min, followed by 100,000 *g* for 60 min, resulting in the isolation of ER (pellet) and cytosolic (supernatant) fractions. The protocol for cultured cells was identical except that BSA was removed from the homogenization buffer. In our experiments, 10 confluent dishes (Φ 15 cm) containing HEK293 cells were sufficient to isolate enough crude mitochondrial fraction.

### 2.7 Immunoprecipitation of AMPD3

Precleared tissue or cell lysates (500 μg) were incubated with 0.64 μg of anti‐AMPD3 antibody (Proteintech 23997‐1‐AP) or 1 μg of rabbit control IgG (Cell Signaling 2729 S) in IP buffer (150 mM NaCl, 10 mM Tris–HCl (pH 7.4), 1 mM EDTA, 1 mM EGTA (pH 8.0), 0.2 mM sodium orthovanadate, 0.2 mM PMSF, 1% Triton X‐100, and 0.5% NP‐40) at 4°C overnight with rotation. The antibody‐AMPD3 complex was collected with magnetic beads (New England Biolabs #S1425S) and washed with IP buffer. The immunoprecipitates were subjected to mass spectrometry or immunoblotting.

### 2.8 Mass spectrometry

Immunoprecipitates obtained with an anti‐AMPD3 antibody or rabbit control IgG were eluted with glycine buffer (0.1 M glycine and 0.02 M HCl, pH 2.5–3.0) and then neutralized by the addition of Tris–HCl (pH 8.5). Subsequent proteomic analyses were performed at the Proteomics Facility at our institute. Briefly, after reduction and alkylation, in‐solution digestion with trypsin was performed overnight at 37°C. Samples were dissolved in 0.1% formic acid and loaded onto a nanoflow ultrahigh‐performance liquid chromatograph column (Easy‐nLC 1000 system, Thermo Fisher Scientific) coupled online to an Orbitrap mass spectrometer equipped with a nanospray ion source (Q‐Exactive Plus, Thermo Fisher Scientific). The samples were separated on a 75 μm × 20 cm capillary column with a particle size of 3 μm (NTCC‐360, Nikkyo Technos) by applying a linear gradient ranging from 5% to 35% buffer B (100% acetonitrile and 0.1% formic acid) at a flow rate of 300 nL/min for 120 min. Through mass spectrometry analysis, survey spectra were acquired at a resolution of 70,000 at 200 m/z with a target value of 3e6 ions, ranging from 350 to 2000 m/z with charge states between 1+ and 4+. We applied a data‐dependent top‐10 method, which generates high‐energy collision dissociation fragments for the 10 most intense precursor ions per survey scan. The tandem mass spectrometry resolution was 17,500 at 200 m/z with a target value of 1e5 ions. The data were searched against the SWISS‐PROT human proteome database using the Sequest HT search engine with Proteome Discoverer 2.2 software (Thermo Fisher Scientific).

### 2.9 Immunoblotting

To obtain total lysates, frozen tissue samples or fresh cultured cells were homogenized in ice‐cold buffer (CelLytic™ MT Cell Lysis Reagent for tissue samples or CelLytic™ M Cell Lysis Reagent for cultured cells, Sigma Aldrich) with protease inhibitor cocktail (NACALAI TESQUE 25955‐11) and phosphatase inhibitor cocktail (NACALAI TESQUE 07574‐61). The homogenate was centrifuged at 13,000 *g* for 15 min at 4°C to obtain the supernatant. Fractionation samples were prepared as described above. Protein concentration was determined using a BCA Protein Assay Kit (TaKaRa Bio T9300A). Equal amounts of proteins (5–10 μg) were electrophoresed on 7.5% or 10% polyacrylamide gels and then blotted onto PVDF membranes. After blocking with a TBS‐T buffer containing 5% nonfat dry milk or 5% BSA, the blots were incubated with primary antibodies at 4°C overnight. The following primary and secondary antibodies were used in this study: anti‐AMPD3 (Proteintech 23997‐1‐AP, 1:1000–1:2000); anti‐BCKDHA (Abcam ab138460, 1:2000–1:4000); anti‐BCKDHA (phospho‐S293) (Abcam ab200577, 1:2000); anti‐DBT (Abcam ab151991, 1:2000); anti‐DLD (Gene Tex GTX101245, 1:2000); anti‐KLF15 (Santa Cruz sc271675, 1:500); anti‐BCAT1 (Proteintech 13640‐1‐AP, 1:2000); anti‐BCAT2 (Proteintech 16417‐1‐AP, 1:1000); anti‐PP2Cm (Proteintech 14573‐1‐AP, 1:500); anti‐BCKDK (Santa Cruz sc374425, 1:100); anti‐AKT (phospho‐S473) (Cell Signaling 9271 S, 1:1000); anti‐AKT (Cell Signaling 9272 S, 1:1000); anti‐CPT1B (Proteintech 22170‐1‐AP, 1:2000); anti‐PGK1 (Gene Tex GTX107614, 1:2000); anti‐calnexin (Abcam ab22595, 1:40000); anti‐SERCA2 ATPase (Abcam ab2861, 1:2500); anti‐cyclophilin D (Gene Tex GTX117951, 1:1000); anti‐COX4 (Santa Cruz sc517553, 1:400); anti‐cytochrome c (Cell Signaling 4272 S, 1:1000); anti‐VDAC1 (Santa Cruz sc390996, 1:2000); anti‐vinculin (Sigma Aldrich V9131, 1:8000); anti‐AMPK (phospho‐T172) (Cell Signaling 2535 S, 1:2000); anti‐AMPK (Cell Signaling 2532 S, 1:2000); anti‐ACC (phospho‐S79) (Cell Signaling 3661 S, 1:1000); anti‐ACC (Cell Signaling 3676 S, 1:1000); anti‐DGAT1(Abcam ab54037, 1:500); anti‐ATGL (Cell Signaling 2138 S, 1:1000); anti‐ATP‐citrate lyase (Cell Signaling 4332 S, 1:2000); anti‐mouse IgG (Cytiva NA931V, 1:5000); anti‐rabbit IgG (Cytiva NA934V, 1:2000); and anti‐rabbit IgG (Light‐Chain Specific) (Cell Signaling 45262, 1:2000). Immunoblotted proteins were visualized by using an Immobilon Western Detection Kit (Millipore WBKLS0500). Image J software (National Institutes of Health) was used for quantifying signals of each blot.

### 2.10 Quantitative RT–PCR

The mRNA levels of AMPD3, PPARα, and CD36 were measured as previously described (Tatekoshi et al., 2018).

### 2.11 Measurement of mitochondrial respiration

The mitochondrial oxygen consumption rate (OCR) was measured with a Seahorse XFe96 Analyzer (Agilent Technologies, Santa Clara, CA). Twenty‐four hours after NRCMs were plated at a density of 30,000 cells per well, siRNA transfection was performed and the cells were incubated for 24 h. After replacement with fresh culture medium containing 0.5 mM L‐carnitine, the cells were incubated for an additional 24 h. One hour before the assay was performed, the culture medium was replaced with an assay medium (Seahorse XF DMEM, Agilent Technologies 103575‐100) supplemented with 2 mM glucose and 0.5 mM L‐carnitine, pH 7.4. Before the assay was performed, BSA‐conjugated palmitate (molar ratio 6:1, final concentration 167 μM; the XF palmitate‐BSA FAO substrate, Agilent Technologies 102720‐100) or control BSA was added to each well. To avoid fatty acid‐induced cardiomyocyte apoptosis (Sparagna et al., 2000), we adopted the low palmitate concentration, which has been validated in previous studies (Angelini et al., 2022; Readnower et al., 2012). OCR was measured as previously described (Igaki et al., 2021). In preliminary experiments using NRCMs, we confirmed that 40 μM etomoxir completely blocked the palmitate‐stimulated increase in OCR. To assess nonfatty acid oxidation, an assay medium supplemented with 5.5 mM glucose, 1 mM pyruvate, and 2 mM glutamine was used instead of the palmitate‐supplemented assay medium. Insulin was added 15 min before assay with a final concentration of 100 nM. OCR was also measured in H9c2 cells 48 h after transfection with either FLAG‐AMPD3 or FLAG‐control.

### 2.12 Lipid droplet assay

A lipid droplet assay was conducted as previously reported with slight modification (Nguyen et al., 2017). Briefly, 24 h after transfection, NRCMs were incubated with fresh culture medium containing different concentrations of BSA‐conjugated oleate (molar ratio 2:1, final concentration 2/10/20 μM; Sigma Aldrich O3008) for 24 h. Before fixation, lipid droplets were stained with BODIPY 493/503 (Thermo Fisher Scientific D3922) for 15 min according to the manufacturer's instructions. Nuclei were stained with Hoechst. Images were obtained by confocal microscopy (ELYRAS.1LSM780, ZEISS) with a 63x oil immersion objective and analyzed using ImageJ software. Images were analyzed on the basis of threshold data, and then, the number and area of BODIPY 493/503‐stained lipid droplets were quantified and normalized on the basis of cell counts. For group comparisons, each experimental group consisted of 6–7 samples, and the lipid droplets in 30–40 cells in each sample were quantified.

### 2.13 Statistical analysis

The data are expressed as the means ± SEM. Differences in metabolite, protein, and mRNA expression and enzyme activity between groups were analysis by Student's 2‐tailed *t*‐test or one‐way ANOVA. Differences in lipid droplet assays were tested by two‐way ANOVA. Mitochondrial respiration assays were tested by two‐way repeated‐measures ANOVA. Tukey–Kramer test was performed for multiple comparisons when ANOVA indicated significant differences. A difference was considered significant when the *p*‐value was <0.05. The analysis was conducted using GraphPad Prism 9 software.

## 3 RESULTS

### 3.1 Downregulation of BCAA catabolic pathway is associated with BCAA accumulation in T2DM hearts

**FIGURE 1:**
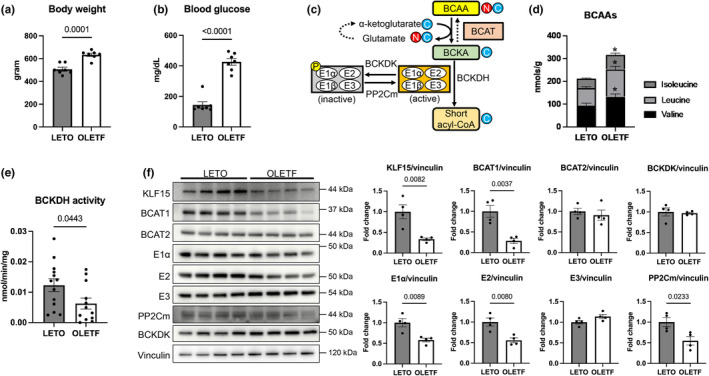
Downregulation of branched‐chain amino acid (BCAA) catabolic pathway is associated with BCAA accumulation in type 2 diabetes (T2DM) hearts. (a, b) Body weight (a; *N* = 7 in each group) and casual blood glucose (b; *N* = 7 in each group) in LETO and OLETF rats. (c) A schematic showing the BCAA degradation pathway. A red circle indicates the amino group in BCAA, and a blue circle indicates its carbon backbone. (d, e) Myocardial BCAA levels (d; *N* = 5 in each group) and BCKDH activity (e; *N* = 12–13 in each group) in LETO and OLETF rats. (f) Representative Western blot showing BCKDH catabolic pathway in the total lysate of LETO and OLETF rat hearts and summary of the densitometry analysis (*N* = 4 in each group). BCAAs, branched‐chain amino acids; BCAT, branched‐chain amino acid aminotransferase; BCKA, branched‐chain keto acid; BCKDH, branched‐chain a‐keto acid dehydrogenase; BCKDK, branched‐chain keto acid dehydrogenase kinase; PP2Cm, mitochondrial matrix‐targeted protein phosphatase 2C family member; KLF15, Krüppel‐like factors 15. Data were analyzed by unpaired Student's *t*‐test. *p < 0.05 between BCAA levels in LETO and OLETF rats. The *p*‐values obtained for comparisons of the groups at both ends of the line are shown.

As we previously described (Hotta et al., 2010; Kouzu et al., 2015; Miki et al., 2009; Tatekoshi et al., 2018), OLETF rats had significantly higher body weight and casual blood glucose than LETO rats (Figure 1a,b), confirming the OLETF rat as an obese T2DM model. BCAA is converted to branched‐chain keto acid (BCKA) through transamination by branched‐chain amino acid aminotransferase (BCAT) and then oxidized by BCKDH. BCKDH activity is inhibited by phosphorylation of its E1α subunit at the serine 293 residue by branched‐chain keto acid dehydrogenase kinase (BCKDK), and dephosphorylation of E1α is realized by mitochondrial matrix‐targeted protein phosphatase 2C family member (PP2Cm), which activates BCKDH (Figure 1c). Myocardial BCAAs were significantly accumulated in OLETF rats compared to LETO rats (Figure 1d), and this accumulation was associated with lower BCKDH activity (Figure 1e), consistent with previous animal models demonstrating impaired cardiac BCAA metabolism and increased tissue concentrations of BCAAs (McGarrah & White, 2022). In addition, elevated circulating BCAAs in OLETF (Kuzuya et al., 2008) may have also contributed to the higher cardiac BCAAs, as the plasma level is another determinant of cardiac BCAAs (McGarrah & White, 2022).

Krüppel‐like factors 15 (KLF15), a transcriptional factor that regulates the gene expressions of the BCAA catabolic pathway (Sun et al., 2016), was significantly downregulated by 66% in OLETF rats (Figure 1f). Consistent with the KLF15 downregulation, the expressions of its downstream targets, including the cytosolic form of BCAT1, E1α, and E2 subunit of BCKDH, and PP2Cm were significantly decreased. Despite the downregulation of PP2Cm, we did not observe a significant difference in the level of E1α phosphorylation between the LETO and OLETF rats (Figure S1). Thus, the suppressed BCKDH activity in OLETF rats appeared to be attributable to the downregulation of BCKDH components rather than posttranslational modification of E1α.

### 3.2 AMPD3 expression negatively regulates BCKDH activity, in which direct interaction of AMPD3 and BCKDH might be involved

**FIGURE 2:**
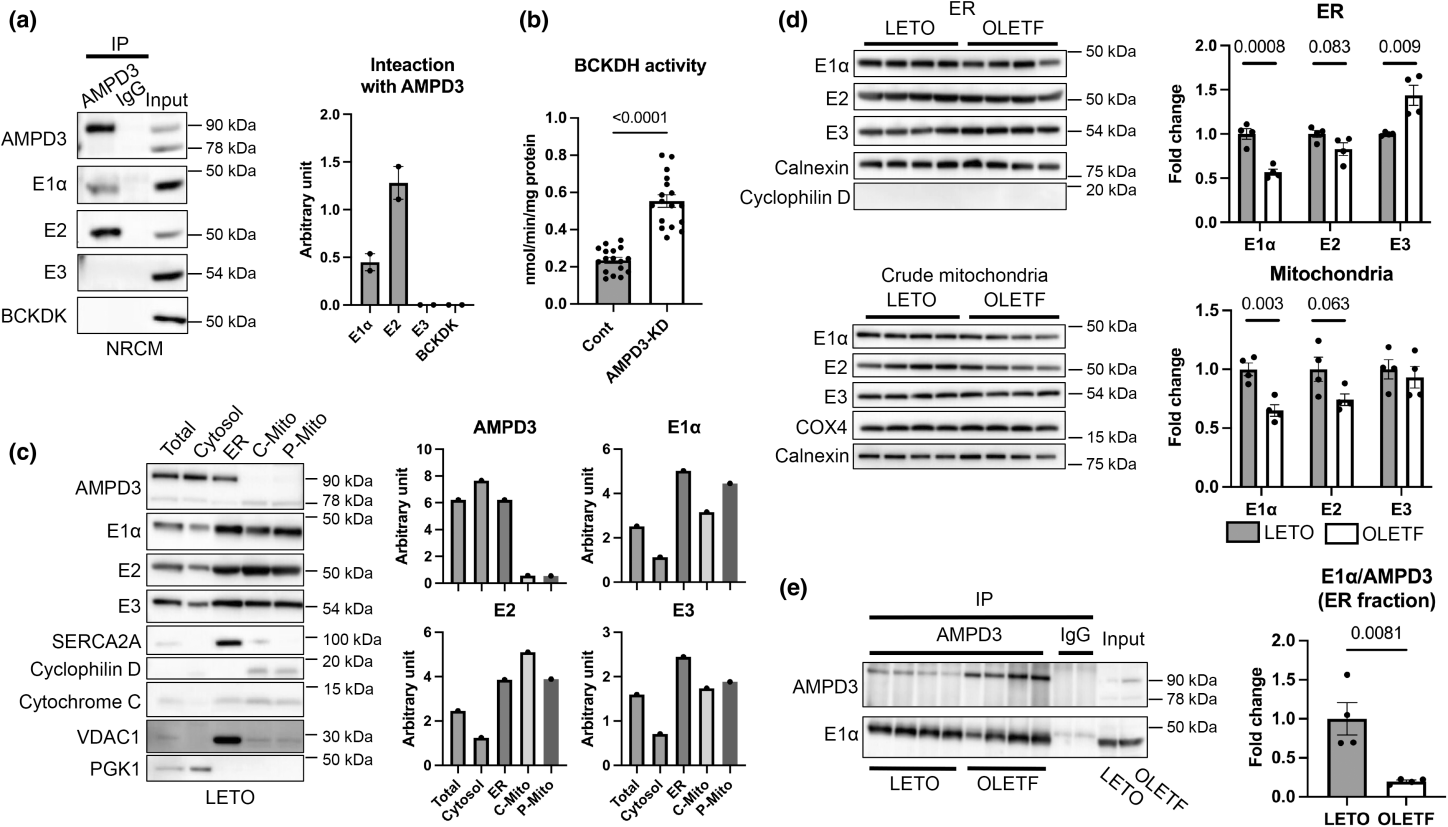
AMP deaminase 3 (AMPD3) expression negatively regulates branched‐chain α‐keto acid dehydrogenase (BCKDH) activity, in which direct interaction of AMPD3 and BCKDH might be involved. (a) Representative Western blot showing the AMPD3‐BCKDH interaction in neonatal rat cardiomyocytes (NRCMs) and summary of the densitometry analysis (*N* = 2). (b) BCKDH activity (*N* = 17 in each group) in NRCMs with or without AMPD3 knocked down. (c) Western blot showing AMPD3 and BCKDH components in subcellular compartments of hearts from LETO rat and summary of the densitometry analysis. (d) Representative Western blot showing BCKDH components in subcellular compartments of LETO and OLETF rat hearts and summary of the densitometry analysis (*N* = 4 in each group). Normalization was performed using the corresponding loading control for each fraction. (e) Representative Western blot showing the AMPD3‐BCKDH interaction in the ER fraction (*N* = 4 in each group) of LETO and OLETF rat hearts and summary of the densitometry analysis. ER, endoplasmic reticulum; C‐Mito, crude mitochondria; P‐Mito, pure mitochondria. Data were analyzed by unpaired Student's *t*‐test. The *p*‐values obtained for comparisons of the groups at both ends of the line are shown.

Our previous proteomic analysis based on two‐dimensional gel electrophoresis indicated that AMPD3 interacts with the E1α subunit of BCKDH in rat cardiac tissues (Tatekoshi et al., 2018). We verified this finding by performing direct proteomic analyses of AMPD3 immunoprecipitates in the LETO and OLETF rat heart lysates (Table S1). The top candidates for binding were the E3 subunit of BCKDH and BCKDK, which binds to the E2 subunit then phosphorylates the E1α subunit (Zhou et al., 2012). These results suggest that AMPD3 interacts with the BCKDH enzyme complex. Next, we validated the interaction by immunoblotting. As we previously reported (Tatekoshi et al., 2018), rat AMPD3 is expressed as 2 isoforms: full‐length AMPD3 (90 kDa) and N‐terminus‐truncated AMPD3 (78 kDa). Full‐length AMPD3 interacted with the E1α and E2 subunits but not with the E3 subunit and BCKDK in NRCMs (Figure 2a). We could not determine whether truncated AMPD3 can interact with BCKDH because the antibody we used did not immunoprecipitate this isoform. Knocking down AMPD3 in NRCMs significantly increased BCKDH activity 2.4‐fold, indicating that AMPD3 negatively regulates BCKDH (Figure 2b). Unexpectedly, the enhanced BCKDH activity by AMPD3 knockdown was not associated with a decrease in E1α phosphorylation or increase in protein levels of E1α, E2, and E3; the subunits of BCKDH were rather reduced by AMPD3 knockdown (Figure S2). Knockdown of E1α expression by 75% reduced BCKDH activity by 80% in H9c2 cells (data not shown), confirming the protein level of BCKDH being a determinant of its activity. These results suggest that there are contribution of a previously unidentified mechanism to the increase in BCKDH activity and negative feedback response of BCKDH expression in cells with knockdown of AMPD3 expression. However, we cannot exclude the possibility that regulatory mechanisms of BCKDH by AMPD3 differ between intracellular compartments.

The interaction of BCKDH with AMPD3 led us to examine the subcellular localization of BCKDH in cardiac tissues because AMPD3 was reported to localize with cytoplasmic membrane (Mahnke‐Zizelman & Sabina, 2002), while BCKDH is a mitochondrial matrix protein complex. As expected, full‐length AMPD3 is predominantly expressed in extramitochondrial compartments obtained from LETO rats (Figure 2c). However, we found that BCKDH components were highly enriched not only in the mitochondrial fraction but also in the ER fraction and, to a lesser extent, in the cytosolic fraction. Proteins localized in the outer mitochondrial membrane (VDAC1) and inner mitochondrial membrane space (cytochrome C) were detected in the ER fraction because the ER and mitochondria structurally interact at the mitochondria‐associated membrane. However, it is unlikely that the ER fraction is heavily contaminated with mitochondrial matrix because there was no signal for cyclophilin D, mitochondrial matrix protein, in the ER fraction with comparable signal intensities for BCKDH in the ER and mitochondria fractions (Figure 2c,d). In addition, AMPD3, which is predominantly localized in extramitochondrial compartments, was found to physically interact with BCKDH (Figure 2a, Table S1). Taken together, it would be reasonable to conclude that BCKDH localizes in not only mitochondria but also extramitochondrial compartments. Extramitochondrial BCKDH expression was not limited to this rat strain; notably, along with AMPD3, BCKDH was also highly expressed in the ER fraction obtained from the heart of Sprague–Dawley rats (Figure S3). We also examined whether AMPD3 interacts with BCKDH in HEK293 cells, which are derived from human embryonic kidney cells. Interestingly, although HEK293 cells showed different BCKDH and full‐length AMPD3 expression patterns compared to those in the myocardium (i.e., BCKDH is expressed exclusively in mitochondria, while full‐length AMPD3 is ubiquitously expressed, including in mitochondria), the interaction of full‐length AMPD3 with the E1α and E2 subunits was also detected in HEK293 cells (Figure S4). These results indicate that intracellular compartments where AMPD3‐BCKDH interaction occurs are different depending on cell types.

In the OLETF rats, E1α expression was decreased by 43% in the ER fraction and 35% in the mitochondrial fraction (Figure 2d). E2 subunit expression also tended to be decreased in the ER and mitochondria fractions and was significantly decreased in the cytosol fraction, while E3 expression in the ER was increased by 43% (Figure 2d, Figure S5a). As expected by their colocalization, AMPD3‐E1α interaction was confirmed in the ER and cytosolic fractions obtained from LETO and OLETF rats (Figure 2e, Figure S5b). On the contrary, full‐length AMPD3 in the pure mitochondrial fraction was difficult to obtain by immunoprecipitation, probably due to its low expression in mitochondria. As we previously reported (Tatekoshi et al., 2018), full‐length AMPD3 expression was upregulated in OLETF rats. The upregulated AMPD3 expression and the downregulation of E1α expression resulted in a significantly lower E1α‐AMPD3 interaction ratio in OLETF than in LETO rats in the ER and cytosolic fractions (Figure 2e, Figure S5b). These results indicate that an imbalanced extramitochondrial AMPD3‐BCKDH interaction may be involved in the suppressed BCKDH activity observed in T2DM hearts.

### 3.3 Reduced BCKDH‐E1α expression upregulates AMPD3

**FIGURE 3:**
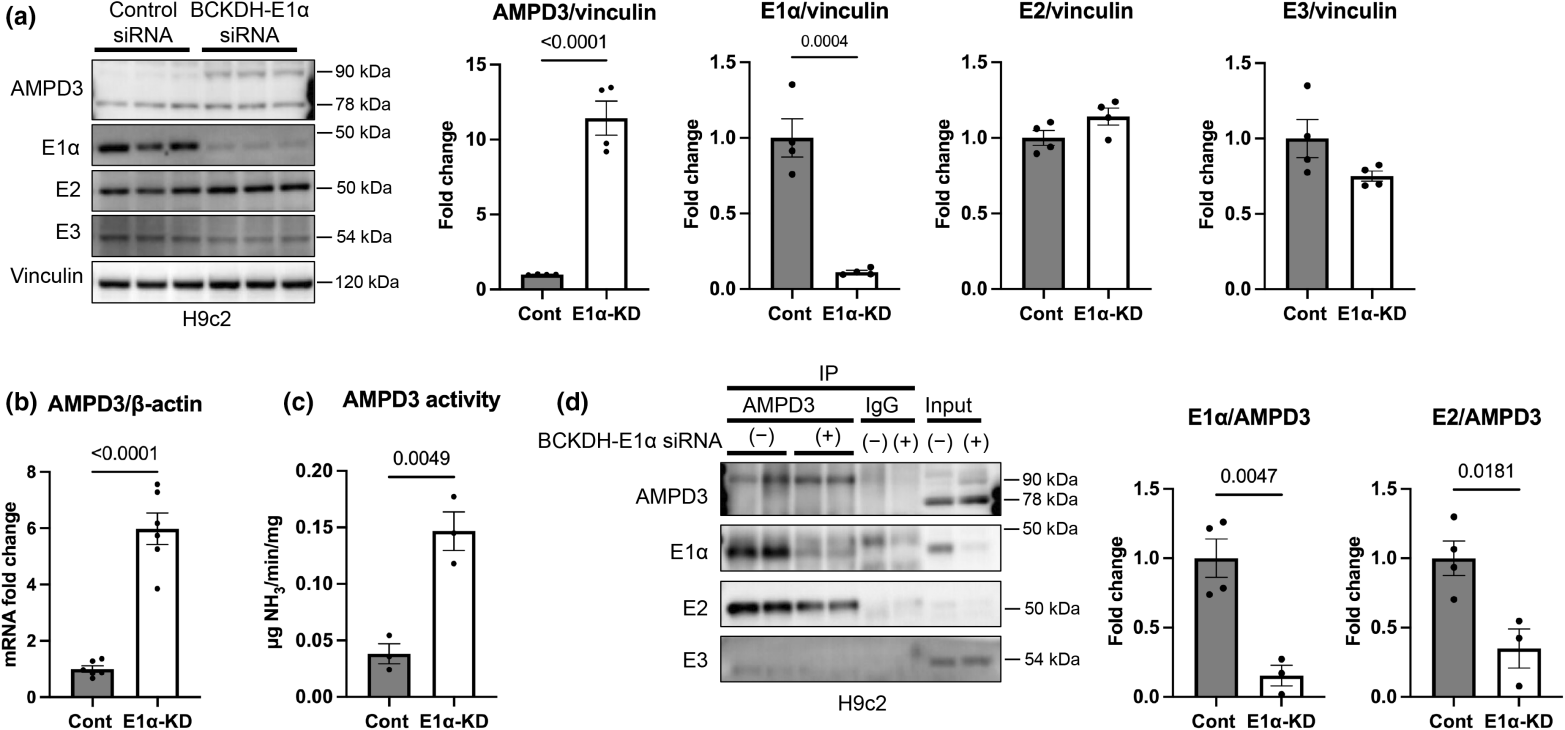
Knockdown of BCKDH‐E1α expression upregulated AMPD3 expression. (a–d) Representative Western blot showing AMPD3, BCKDH‐E1α, E2 and E3, and summary of the densitometry analysis (a; *N* = 4 in each group); mRNA levels of AMPD3 (b; *N* = 6 in each group); AMPD3 activity (c; *N* = 3 in each group); and a representative Western blot showing the AMPD3‐BCKDH interaction and summary of the densitometry analysis (d; *N* = 3–4 in each group) in H9c2 cardiomyoblasts with or without E1α knocked down. Data were analyzed by unpaired Student's *t*‐test. The *p*‐values obtained for comparisons of the groups at both ends of the line are shown.

We next examined whether the downregulated expression of BCKDH components is causally linked to the upregulated expression of AMPD3 observed in OLETF rats. In H9c2 cardiomyoblasts, disruption of BCAA metabolism by knocking down E1α subunit expression upregulated full‐length AMPD3 protein expression more than 10‐fold and AMPD3 mRNA expression more than 6‐fold, and AMPD3 deamination activity was increased 3.8‐fold (Figure 3a–c). Knocking down E1α expression did not affect E2 and E3 subunit expression levels (Figure 3a). Consistent with the findings with NRCMs, the analysis in H9c2 cells revealed that full‐length AMPD3 interacted with the E1α and E2 subunits but not with the E3 subunit, resulting in a significantly lower AMPD3 interaction with E1α and E2 in cells with E1α knockdown (Figure 3d). Taken together with the changes in BCAA metabolic pathway observed in OLETF hearts (Figure 1f), the results suggest that the downregulation of BCKDH expression is upstream of AMPD3 upregulation, which further suppresses BCKDH activity in T2DM hearts.

### 3.4 Reduction in BCKDH‐E1α expression level suppresses fatty acid oxidation and insulin‐mediated glucose oxidation in cardiomyocytes

**FIGURE 4:**
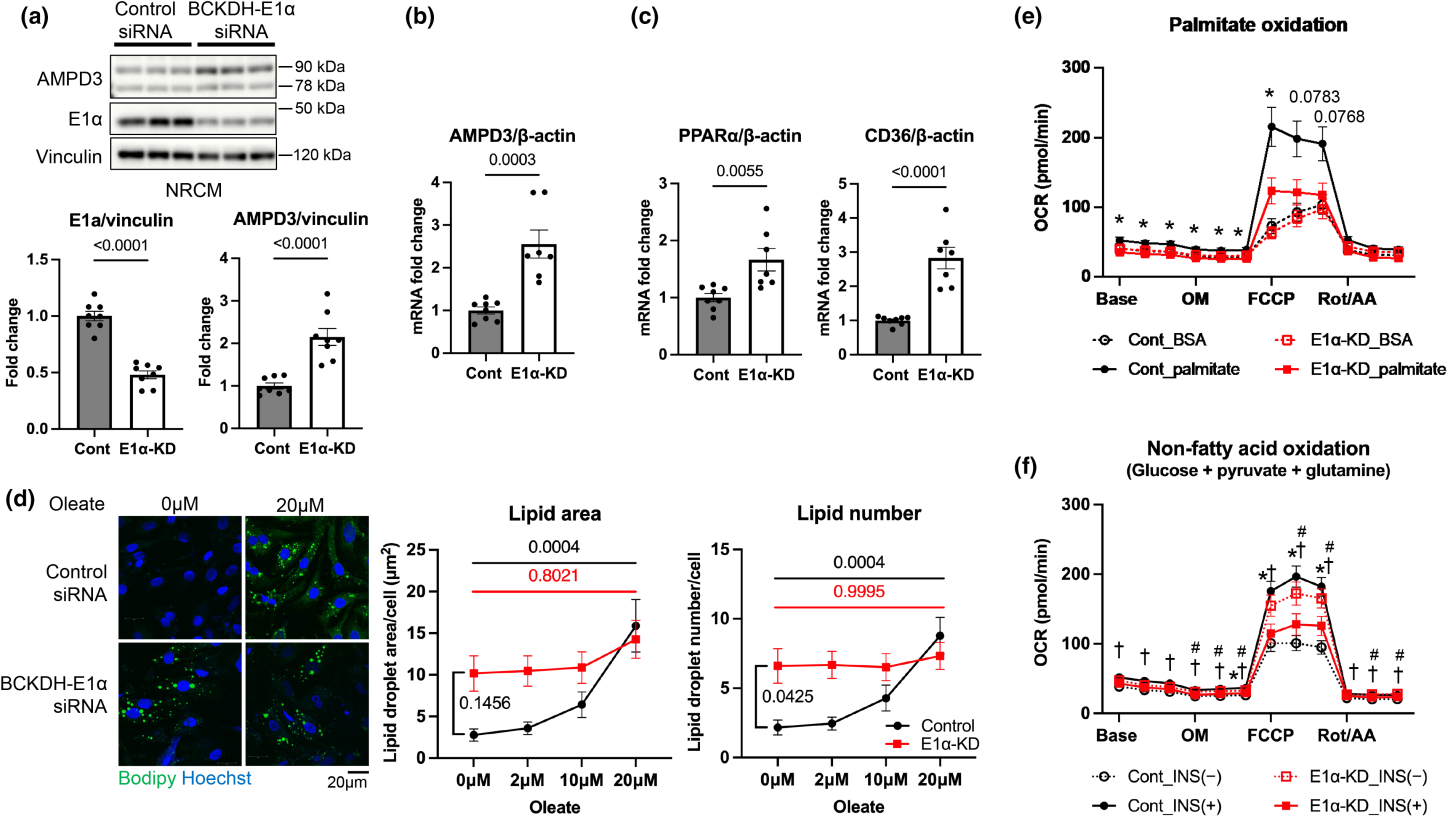
Effects of knockdown of BCKDH‐E1α protein expression on fatty acid oxidation and insulin‐mediated glucose oxidation in cardiomyocytes. (a, b) Representative Western blot showing AMPD3 and BCKDH‐E1α and summary of the densitometry results (a; *N* = 8 in each group) and mRNA levels of AMPD3 (b; *N* = 7–8 in each group) in NRCMs with or without E1α knocked down. (c) mRNA levels of PPARα and CD36 (*N* = 7–8 in each group) in NRCMs with or without E1α knocked down. (d) Representative image showing intracellular lipid droplets and summary of their quantification in NRCMs with or without E1α knocked down. For each dose of oleate, *N* = 6–7 for each group. (e, f) Oxygen consumption rate (OCR) in response to fatty acid (e; *N* = 19–27 in each group) or nonfatty acid (f; *N* = 12–21 in each group) substrates in NRCMs with or without E1α knocked down, as determined with a Seahorse XFe96 analyzer. The concentrations of each reagent were OM, 1.5 μM, FCCP, 1.5 μM, Rot, 0.5 μM, AA, 0.5 μM, BSA‐conjugated palmitate (molar ratio 6:1), 167 μM, and INS, 100 nM. Base, baseline; BSA, bovine serum albumin; OM, oligomycin; FCCP, carbonylcyanide‐4‐trifluoromethoxyphenylhydrazone; Rot, rotenone; AA, antimycin; INS, insulin. Data were analyzed by unpaired Student's *t*‐test (a‐c), 2‐way ANOVA (D) or two‐way repeated‐measures ANOVA (e, f) with Tukey's test for multiple‐group comparison. *p < 0.05 between Cont_palmitate and E1α‐KD_palmitate in (e). *p < 0.05 between Cont_INS(+) and E1α‐KD_INS(+), ^†^ *p* < 0.05 between Cont_INS(−) and Cont_INS(+), ^#^ *p* < 0.05 between Cont_INS(−) and E1α‐KD_INS(−) in (f). The *p*‐values obtained for comparisons of the groups at both ends of the line or between Cont_palmitate and E1α‐KD_palmitate are shown.

Previous studies showed that the accumulation of BCAA (Li et al., 2017) or BCKA (Uddin et al., 2021) suppressed glucose oxidation in the mouse heart. We hypothesized that disruption of the BCAA catabolic pathway also affects cardiomyocyte fatty acid metabolism based on the regulation of BCKDH by AMPD3 (Figure 2, Figure 3) and on the previous study demonstrating that AMPD3 mediates the temporal alignment of BCAA and fatty acid metabolism in skeletal muscle (Hong et al., 2017). In NRCMs, knocking down E1α expression upregulated AMPD3 expression more than 2‐fold at protein and mRNA levels (Figure 4a,b), consistent with the finding with H9c2 cells. E1α knockdown also increased the gene expression of PPARα and membrane fatty acid transporter CD36 (Figure 4c), suggesting that BCAA dysmetabolism promotes substrate preference towards fatty acids. Once fatty acids enter cells, they are incorporated into lipid droplets, which serve as efficient reservoirs for mitochondrial oxidation (Banke et al., 2010) and as essential sources of lipid ligands for PPARα activation (Haemmerle et al., 2011). Our previous study revealed that myocardial lipid droplets are significantly increased in OLETF than in LETO (Mizuno et al., 2018). To test whether BCAA dysmetabolism is directly linked to the altered lipid droplet biogenesis, we next performed an in vitro lipid droplet assay. When NRCMs were incubated with different doses of oleate, the control cells exhibited increased lipid droplet formation in a dose‐dependent manner. However, the cells in which E1α expression had been knocked down exhibited a higher number of lipid droplets even before oleate incubation, and oleate‐induced lipid droplet biogenesis was abolished in these cells (Figure 4d), indicating that intact BCAA metabolism is required for physiological lipid storage response in cardiomyocytes.

Inhibition of BCKDH activity by PP2Cm deletion was shown to inhibit pyruvate‐stimulated complex I respiration but not palmitoyl‐carnitine‐stimulated complex I respiration in isolated cardiac mitochondria (Li et al., 2017), seemingly indicating that the impact of BCAA dysmetabolism on mitochondrial respiration is specific to carbohydrate oxidation. However, experiments on isolated mitochondria cannot elucidate the significance of extramitochondrial BCKDH, which interacts with AMPD3. Therefore, we conducted a mitochondrial respiration assay in beating NRCMs. The addition of palmitate to the glucose‐limited assay buffer significantly increased OCR, which was blocked by treatment with etomoxir, a carnitine palmitoyltransferase 1 (CPT I) inhibitor, confirming that NRCMs can utilize palmitates for oxidative phosphorylation (Figure S6). Contrary to the previous finding in isolated mitochondria (Li et al., 2017), palmitate oxidation was significantly decreased by E1α knockdown despite the upregulation of PPARα (Figure 4e). When the respiration assay was performed under non‐fatty acid substrate (mainly glucose) incubation, E1α knockdown tended to increase OCR (Figure 4f). The addition of insulin to this assay medium significantly enhanced OCR in control cells. However, the effect of insulin on OCR was abolished by E1α knockdown, despite the comparable level of AKT phosphorylation to control cells (Figure S7). Overexpression of AMPD3 in H9c2 cells mirrored the effect of E1α knockdown on OCR (Figure S8), suggesting that the upregulation of AMPD3 also contributes to impaired substrate oxidation in cells with BCAA dysmetabolism. Taken together, the results indicate that intact BCAA metabolism is required for efficient fatty acid oxidation as well as insulin‐mediated glucose oxidation in cardiomyocytes.

### 3.5 Reduction in BCKDH‐E1α expression level induced discordant upregulation of enzymes regulating fatty acid metabolism

**FIGURE 5:**
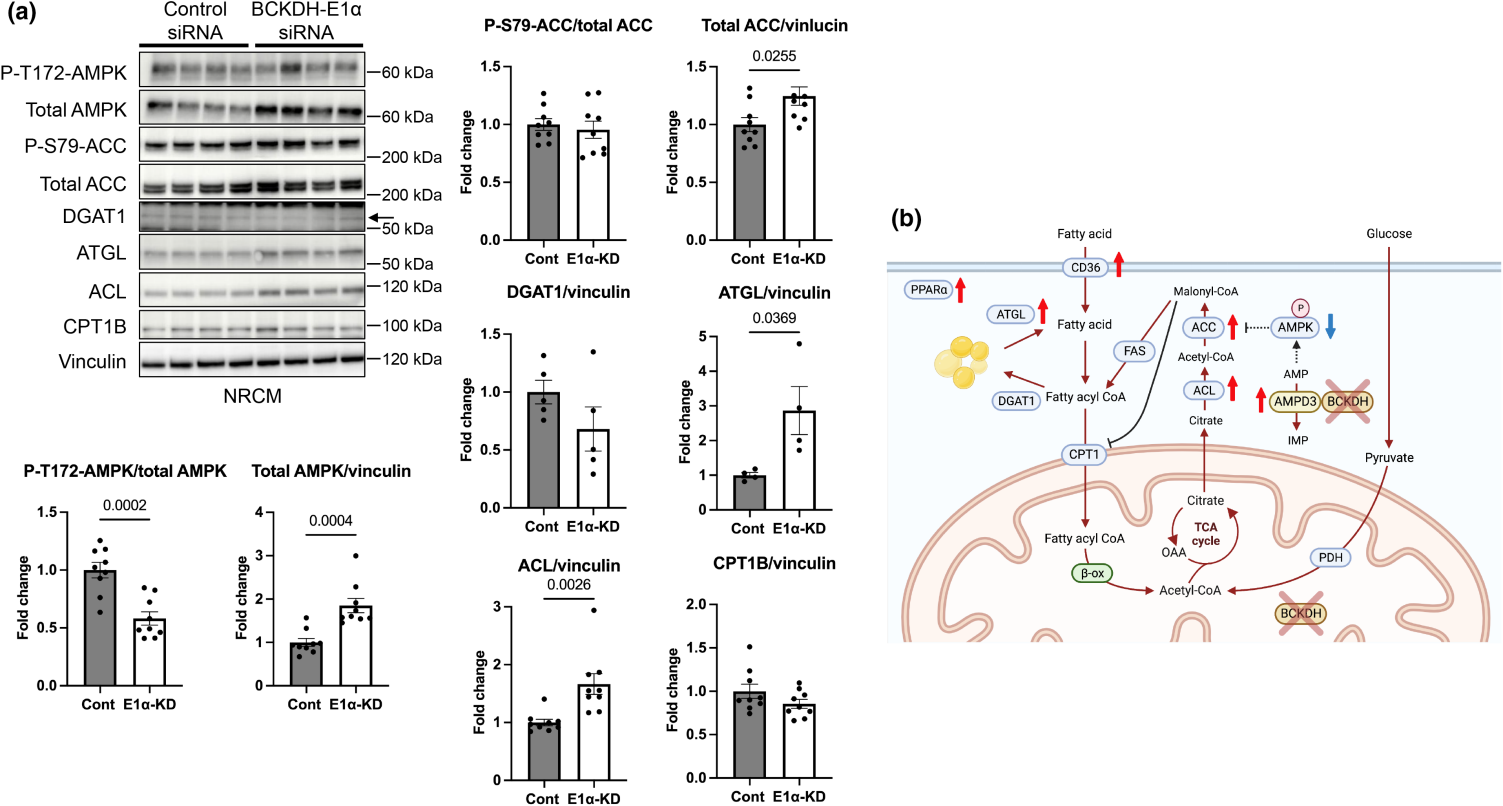
Reduction in BCKDH‐E1α expression level induced discordant upregulation of enzymes regulating fatty acid metabolism. (a) Representative Western blot showing the pathway upstream of mitochondrial fatty acid import and summary of the densitometry analysis in NRCMs with or without E1α knocked down (*N* = 4–9 in each group). (b) A schematic summary showing the changes induced by E1α knockdown in NRCMs. ACL, ATP‐citrate lyase; ACC, acetyl‐CoA carboxylase; CPT1, carnitine palmitoyltransferase 1; DGAT1, diacylglycerol O‐acyltransferase 1; FAS, fatty acid synthase. Data were analyzed by unpaired Student's *t*‐test. The *p*‐values obtained for comparisons of the groups at both ends of the line are shown.

To deduce the mechanisms by which fatty acid oxidation was suppressed by E1α knockdown despite PPARα and CD36 upregulation, we additionally examined changes in the expression of enzymes regulating fatty acid metabolism (Figure 5a). We focused on those upstream of mitochondrial fatty acid import because BCKDH inhibition reportedly did not affect palmitoyl‐carnitine‐stimulated respiration in isolated cardiac mitochondria (Li et al., 2017). E1α knockdown upregulated ATGL, a lipase that liberates fatty acids from lipid droplets as PPARα ligands (Haemmerle et al., 2011), but not DGAT1, consistent with the substrate preference towards fatty acids as mentioned above. However, E1α knockdown simultaneously upregulated ATP‐citrate lyase (ACL) and acetyl‐CoA carboxylase (ACC), likely leading to enhanced production of malonyl‐CoA, a potent endogenous inhibitor of CPT1. Furthermore, the proportion of phosphorylated AMPK, which inhibits ACC activity, was significantly decreased by E1α knockdown.

## 4 DISCUSSION

In this study, we showed, for the first time, that BCKDH is expressed not only in the mitochondrial matrix but also in extramitochondrial compartments, especially the ER in the heart, and that its expression level is downregulated in diabetes. The downregulation of BCKDH in diabetic hearts is likely to be due to the decreased expression of KLF15, a transcriptional factor regulating BCAA catabolic pathway (Sun et al., 2016). Our finding of KLF15 downregulation in OLETF hearts was consistent with earlier reports for adipose tissue and skeletal muscle of insulin‐resistant individuals (Elbein et al., 2011) and rodent diabetic hearts (Tian et al., 2021). BCAA metabolism has long been considered to occur in the mitochondrial matrix, but several lines of recent evidence support the possibility that it also occurs in extramitochondrial compartments. First, although pharmacological activation of BCKDH by the BCKDK inhibitor BT2 has consistently shown to decrease cardiac BCAA levels and improve cardiac function in several models of heart failure (Chen et al., 2019; Li et al., 2017; Sun et al., 2016; Uddin et al., 2019; Wang et al., 2016), an in vivo isotopic tracing study in mice after [U‐^13^C]‐isoleucine infusion showed that BT2 treatment led to only modest increases in the levels of labeled tricarboxylic acid cycle intermediates in the heart (Neinast, Jang, et al., 2019). Second, among the major organs that metabolize BCAAs, the expression of SLC25A44, a mitochondrial BCAA transporter, was the lowest in the myocardium (Walejko et al., 2021). To be oxidized by BCKDH, BCAAs first undergo transamination by cytosolic BCAT1 or mitochondrial BCAT2. BCAT2 is the dominant isoform expressed in the myocardium, but BCAT1 is also functional (Walejko et al., 2021). In addition, circulating BCKAs are directly taken up by the heart (Murashige et al., 2020). These findings suggest that extramitochondrial BCKDH is supplied with its substrates and that the resulting short‐chain acyl‐CoA is consumed for mitochondrial oxidation and also for other metabolic pathways.

Notably, pyruvate dehydrogenase and α‐ketoglutarate dehydrogenase, which share a common E3 subunit with BCKDH, had originally been thought to localize exclusively in mitochondria but were later found to function also in the nucleus (Sutendra et al., 2014; Wang et al., 2017), supporting the possibility that BCDKH functions in extramitochondrial compartments, similar to pyruvate dehydrogenase and α‐ketoglutarate dehydrogenase. A recent study showed that short‐branched‐chain acyl‐CoAs, direct products of the BCKDH reaction, can escape oxidation and be incorporated into newly synthesized fatty acids in adipocytes (Wallace et al., 2018), suggesting a role for BCAAs as lipid metabolite sources. Further studies are warranted to test whether such a metabolic reaction also occurs in cardiomyocytes or whether BCKDH has noncanonical functions in extramitochondrial compartments.

As another novel finding, this study revealed that AMPD3 directly interacts with extramitochondrial BCKDH in the heart. Knocking down AMPD3 significantly increased the whole cell BCKDH activity in living cardiomyocytes (Figure 2b). It remains to be determined whether extramitochondrial BCKDH has physiological activity and is directly inhibited by AMPD3 binding or whether AMPD3 indirectly suppresses mitochondrial BCKDH. In addition to inhibitory phosphorylation of E1α, BCKDH activity is likely to be regulated by its protein abundance because cardiac KLF15 expression is upregulated during fasting when BCAAs are actively metabolized (Murashige et al., 2020; Prosdocimo et al., 2014). However, despite the enhanced BCKDH activity, knocking down AMPD3 did not change E1α phosphorylation status and instead decreased BCKDH expression (Figure S2), indicating the existence of a mechanism independent of these canonical regulations. Indeed, a previous study demonstrated that in vivo tissue BCAA flux does not necessarily correlate with its BCKDH expression level and E1α phosphorylation (Neinast, Jang, et al., 2019). It is tempting to speculate that extramitochondrial BCKDH has physiological activity with a distinct regulatory mechanism and that its aberrant activation induces compensatory feedback inhibition on the canonical regulation by BCKDH protein abundance.

Physiologically, the involvement of AMPD3 in BCAA metabolism seems plausible, given its potential role in intracellular nitrogen homeostasis. In the heart, the carbon skeleton of catabolizing amino acids is fed into metabolic pathways, including the tricarboxylic acid cycle, while their nitrogen is transferred to other amino acids and excreted (Murashige et al., 2020). Such amino acid interconversions could be driven by transamination of BCAA by BCAT and the deamination of AMP by AMPD as they predominantly donate nitrogen utilized for the process (Arinze, 2005). Thus, BCAA metabolism and AMPD3‐mediated reaction are expected to cooperate when amino acid metabolism is activated. Indeed, overexpression of AMPD3 in C2C12 cells reportedly activated the BCAT forward reaction (Miller et al., 2021). If it is also the case in cardiomyocytes, a question is why AMPD3 simultaneously suppresses BCKDH activity while it promotes the BCAT forward reaction. Since BCAAs are unique amino acids that also function as signaling molecules in various biological processes (Neinast, Murashige, & Arany, 2019), their excess degradation might be prevented by AMPD3.

Aberrant upregulation of AMPD3 and its uncoupling with BCKDH appear to be detrimental to energetic metabolism in cardiomyocytes. In skeletal muscle, the expression of AMPD3 is increased by the induction of muscle atrophy, such as fasting (Hong et al., 2017; Lecker et al., 2004; Milan et al., 2015), tumor development (Lecker et al., 2004), renal failure (Lecker et al., 2004), diabetes (Lecker et al., 2004), denervation (Milan et al., 2015), aging (Ibebunjo et al., 2013), and muscle unloading (Brocca et al., 2017). As discussed above, the upregulation of AMDP3 during muscle breakdown seems reasonable, given its role in amino acid catabolism. On the other hand, the uncoupling of BCAA metabolism and AMPD3 activation could hamper cellular energetics. For IMP resulting from AMP deamination to be recycled as adenine nucleotides without degradation, aspartate flux into the purine nucleotide cycle must be maintained (Arinze, 2005). However, aspartate synthesis from oxaloacetate in muscle is impaired under BCAA catabolic dysfunction because the reaction indirectly requires BCAA transamination. As a result, adenine nucleotides are more susceptible to degradation (She et al., 2010).

How AMPD3 is upregulated by suppression of BCKDH expression has yet to be determined. We previously reported that AMPD3 expression was regulated post‐transcriptionally by miR301b (Tatekoshi et al., 2018). However, E1α expression knockdown did not alter miR301b expression (data not shown), suggesting that distinct mechanisms are involved in the altered AMPD3 expression. In skeletal muscle, transcriptional factors FoxO 1/3/4 positively regulated AMPD3 transcription during fasting and hindlimb unloading (Brocca et al., 2017; Milan et al., 2015), while the clock molecule Rev‐erb‐mediated recruitment of histone deacetylase 3 to an enhancer region suppressed AMPD3 transcription during feeding (Hong et al., 2017). Another recent study also demonstrated that AMPD3 transcription is activated by cyclin‐dependent kinase (CDK) 7‐mediated assembly of super‐enhancer in renal tubular epithelial cells and contributes to the development of autosomal dominant polycystic kidney disease (Mi et al., 2020). Contributions of the FoxO‐ and CDK‐7‐mediated mechanisms in AMPD3 upregulation cardiomyocytes remain to be examined.

We showed that manipulation of BCKDH modulates glucose and fatty acid oxidation, the primary two substrates for cardiomyocytes (Figure 4e,f). Earlier studies have demonstrated the crosstalk between glucose and BCAA metabolism in the heart, in which stimulation of glucose uptake inhibits BCAA oxidation and promotes anabolic processes (Shao et al., 2018; Walejko et al., 2021), while BCAA (Li et al., 2017) or BCKA (Uddin et al., 2021) accumulation brakes glucose metabolism. On the contrary, the myocardium is known to simultaneously utilize fatty acids and BCAAs as fuel during fasting (Murashige et al., 2020). The metabolism of BCAA and fatty acids seems to be functionally related because previous studies showed that KLF15 regulates not only the BCAA catabolic pathway but also PPARα activity and fatty acid metabolism in cardiomyocytes (Prosdocimo et al., 2014; 2015).

In the present study, suppression of fatty acid oxidation by knockdown of E1α (Figure 4) was associated with interesting changes in expressions of molecules that regulate fatty acid metabolism (Figure 5). E1α knockdown increased the expressions of PPARα, CD36 (a target of PPARα), and ATGL, indicating increase in substrate preference towards fatty acids (Figure 5). The expression of PPARα (Kyriazis et al., 2021) and ATGL (Mori et al., 2014) in cardiomyocytes is reportedly regulated by FoxO1, a downstream target of AKT. Thus, induction of insulin resistance by impaired BCAA metabolism may partly contribute to aberrant upregulation of PPARα and ATGL in cardiomyocytes. On the contrary, ACL and ACC expressions were increased by knockdown of E1α. The upregulation of the two enzymes would inhibit fatty acid oxidation by inhibiting CPT1 through malonyl‐CoA production (Poirier et al., 2002). Thus, E1α knockdown appears to have induced futile fatty acid handling in which fatty acid availability in the cytosol is increased but their import into the mitochondria is inhibited at a level of CPT1.

The upregulation of ACL and ACC may also explain why lipid droplet formation was augmented in NRCMs with E1α knockdown even before oleate incubation, though change in de novo lipogenesis from glucose was not assessed. Interestingly, in the liver, the transcription factor ChREBP regulates ACL and ACC gene expression cooperatively as a lipogenic gene cluster, while it simultaneously regulates BCKDH activity by modulating gene expression of BCKDK and PP2Cm (White et al., 2018). Whether such a ChREBP‐mediated link between BCKDH and the ACL‐ACC pathway is also present in cardiomyocytes remains to be examined, but it seems an intriguing hypothesis. E1α knockdown also reduced the ratio of phospho‐AMPK, a canonical inhibitor of ACC, to total AMPK. Taken together, findings by BCKDH‐E1α knockdown support the notion that BCKDH plays a significant role in regulation of molecules responsible for transport of fatty acids to mitochondria and their oxidation possibly via interaction with AMPD3.

**FIGURE 6:**
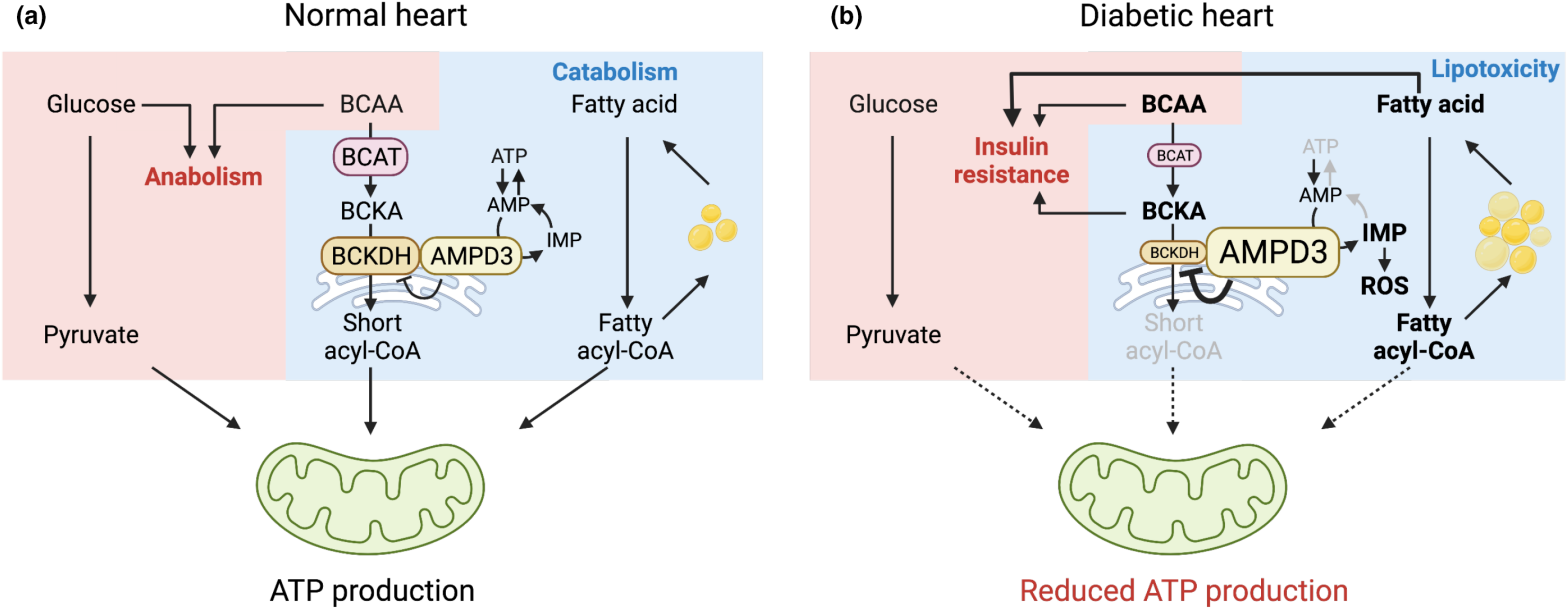
Working model for the functions of the AMPD3‐BCKDH interaction in normal and diabetic hearts based on studies using diabetic OLETF rats. (a) In normal cardiomyocytes, BCAAs, and fatty acids are simultaneously metabolized when glucose availability is limited. In BCAA catabolic pathway, extramitochondrial BCKDH interacts with AMPD3. AMPD3 fine‐tunes BCKDH activity. BCAA catabolism complements efficient fatty acid oxidation. (b) Downregulation of BCKDH expression upregulates AMPD3 expression, which further suppresses BCAA catabolic pathway. The uncoupling of BCAA metabolism and AMPD3 activation causes excessive adenine nucleotide degradation (ref. [Kouzu et al., 2015; Tatekoshi et al., 2018]) and subsequent reactive oxygen species (ROS) production (ref. [Igaki et al., 2021]). The disrupted BCAA metabolism compromises physiological lipid droplet biogenesis and fatty acid oxidation. Glucose oxidation is also disturbed by the accumulation of BCAA/BCKA. These metabolic abnormalities mimic the changes seen in diabetic hearts. The figure was created with BioRender.com.

In conclusion, the present study revealed previously unrecognized extramitochondrial localization of BCKDH in the heart and its reciprocal regulation with AMPD3 and found the imbalance in AMPD3‐BCKDH interaction as a feature of diabetic OLETF rat hearts. Downregulation of BCKDH expression in cardiomyocytes induced profound metabolic changes that mimic OLETF rat hearts. Together with a series of our studies on the roles of AMPD3 in dysregulated cardiac energetics and excessive production of reactive oxygen species in diabetic hearts (Igaki et al., 2021; Kouzu et al., 2015; Tatekoshi et al., 2018), the present findings provide insights into mechanisms contributing to the development of diabetic cardiomyopathy (Figure 6).

## AUTHOR CONTRIBUTIONS

Toshifumi Ogawa contributed to the methodology, validation, formal analysis, and investigation. Hidemichi Kouzu contributed to the conceptualization, methodology, formal analysis, investigation, visualization, writing—original draft, and funding acquisition. Arata Osanami contributed to the methodology, formal analysis, and investigation. Tatekoshi Yuki and Tatsuya Sato contributed to the formal analysis and investigation. Atsushi Kuno contributed to the investigation and supervision. Yugo Fujita, Shoya Ino, Masaki Shimizu, Yuki Toda, and Wataru Ohwada contributed to the investigation. Toshiyuki Yano, Masaya Tanno contributed to the supervision. Takayuki Miki contributed to the data curation and supervision. Tetsuji Miura contributed to the conceptualization, methodology, resources, writing—review and editing, supervision, project administration, and funding acquisition.

## FUNDING INFORMATION

This study was supported by Grant 20 K08452 (H.K.) from the Japan Society for the Promotion of Science, a grant from MSD Life Science Foundation, Public Interest Incorporated Foundation (H.K.).

## CONFLICT OF INTEREST STATEMENT

None.

## ETHICS STATEMENT

Animal studies were conducted according to the Guide for the Care and Use of Laboratory Animals published by the National Research Council of the National Academies, USA (2011) and was approved by the Animal Use Committee of Sapporo Medical University.

## Supporting information


Figure S1.

Figure S2.

Figure S3.

Figure S4.

Figure S5.

Figure S6.

Figure S7.

Figure S8.

Table S1.
