# Supplementary material for: Downregulation of extramitochondrial BCKDH and its uncoupling from AMP deaminase in type 2 diabetic OLETF rat hearts
Source: Physiol Rep. 2023 Feb 17;11(4):e15608. doi: 10.14814/phy2.15608 (PMC9938007; doi:10.14814/phy2.15608)
Supplement: Supplementary file 1 — Figure S1. [file PHY2-11-e15608-s003.pdf]

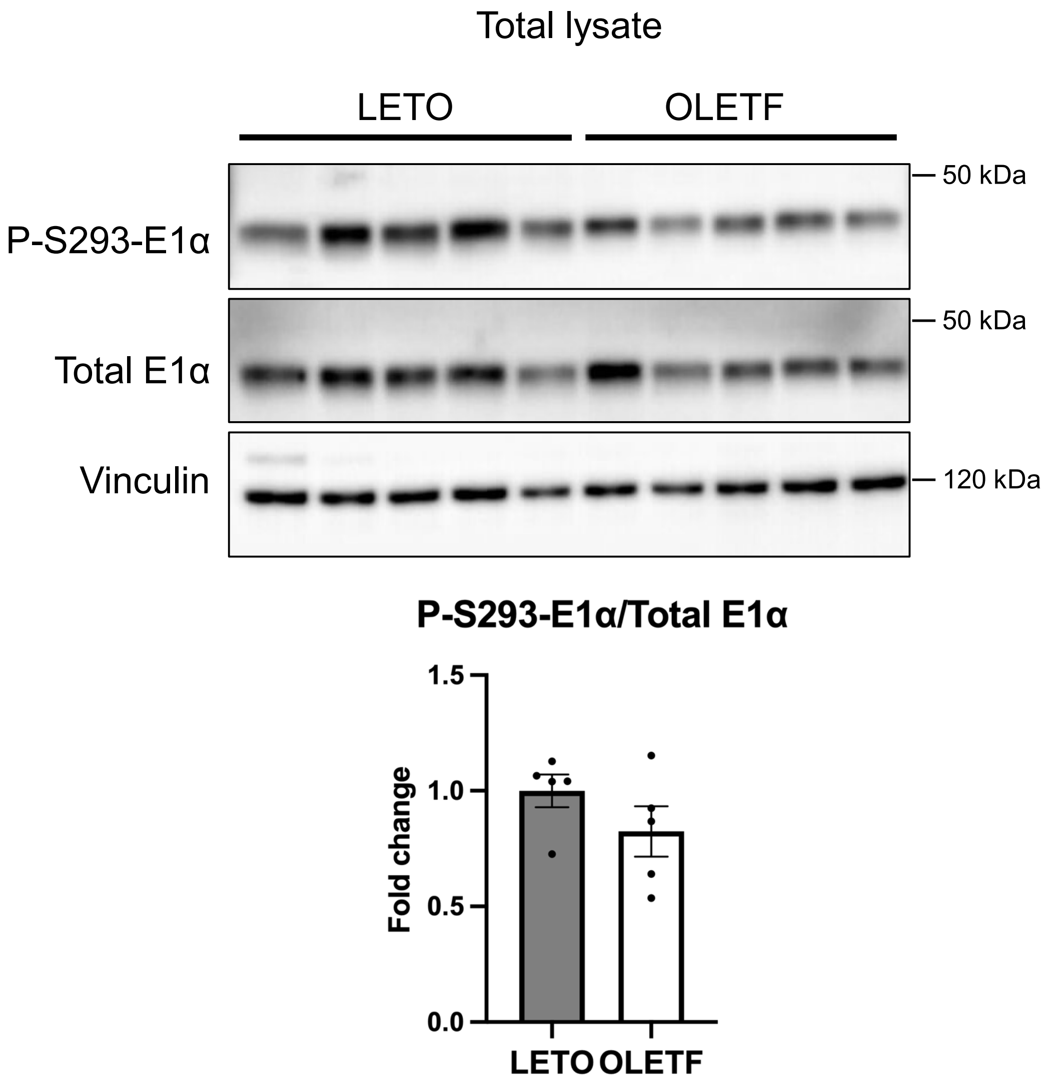

Supplementary Fig. S1. Phosphorylation of BCKDH-E1α in LETO and OLETF (N=5 in each group). Data were analyzed by unpaired Student's t test. P-Ser293-E1α/Total E1α ratios were not significantly different between LETO and OLETF.
