# Supplementary material for: Downregulation of extramitochondrial BCKDH and its uncoupling from AMP deaminase in type 2 diabetic OLETF rat hearts
Source: Physiol Rep. 2023 Feb 17;11(4):e15608. doi: 10.14814/phy2.15608 (PMC9938007; doi:10.14814/phy2.15608)
Supplement: Supplementary file 2 — Figure S2. [file PHY2-11-e15608-s004.pdf]

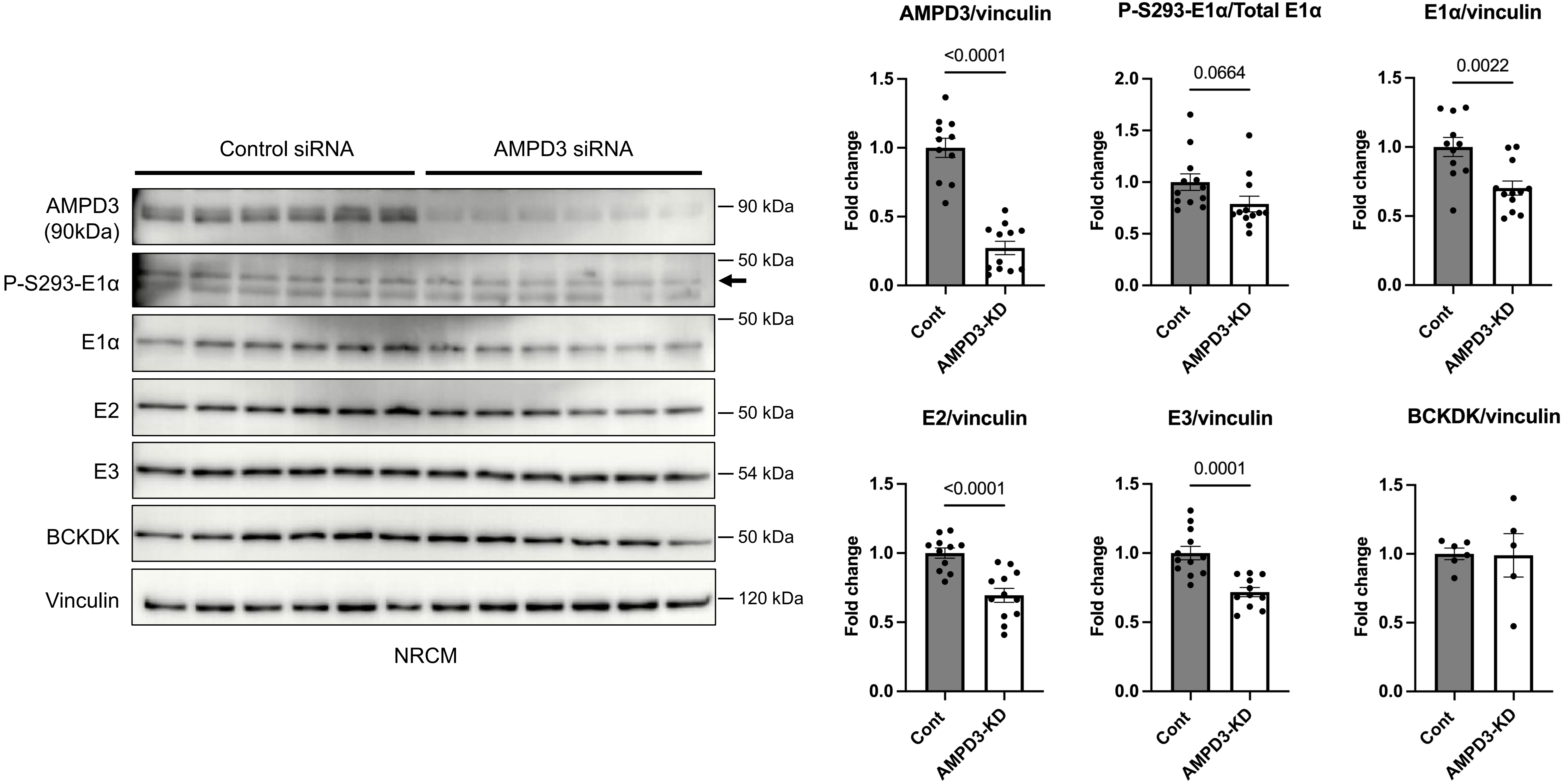

Supplementary Fig. S2. Changes in the expression of BCKDH complex and phosphorylation of E1α in NRCMs with or without AMPD3 knockdown (N=5-12 in each group). The arrow indicates the P-S293-E1α band. Data were analyzed by unpaired Student's t test. The p values obtained for comparisons of the groups at both ends of the line are shown.
