# Supplementary material for: Downregulation of extramitochondrial BCKDH and its uncoupling from AMP deaminase in type 2 diabetic OLETF rat hearts
Source: Physiol Rep. 2023 Feb 17;11(4):e15608. doi: 10.14814/phy2.15608 (PMC9938007; doi:10.14814/phy2.15608)
Supplement: Supplementary file 3 — Figure S3. [file PHY2-11-e15608-s009.pdf]

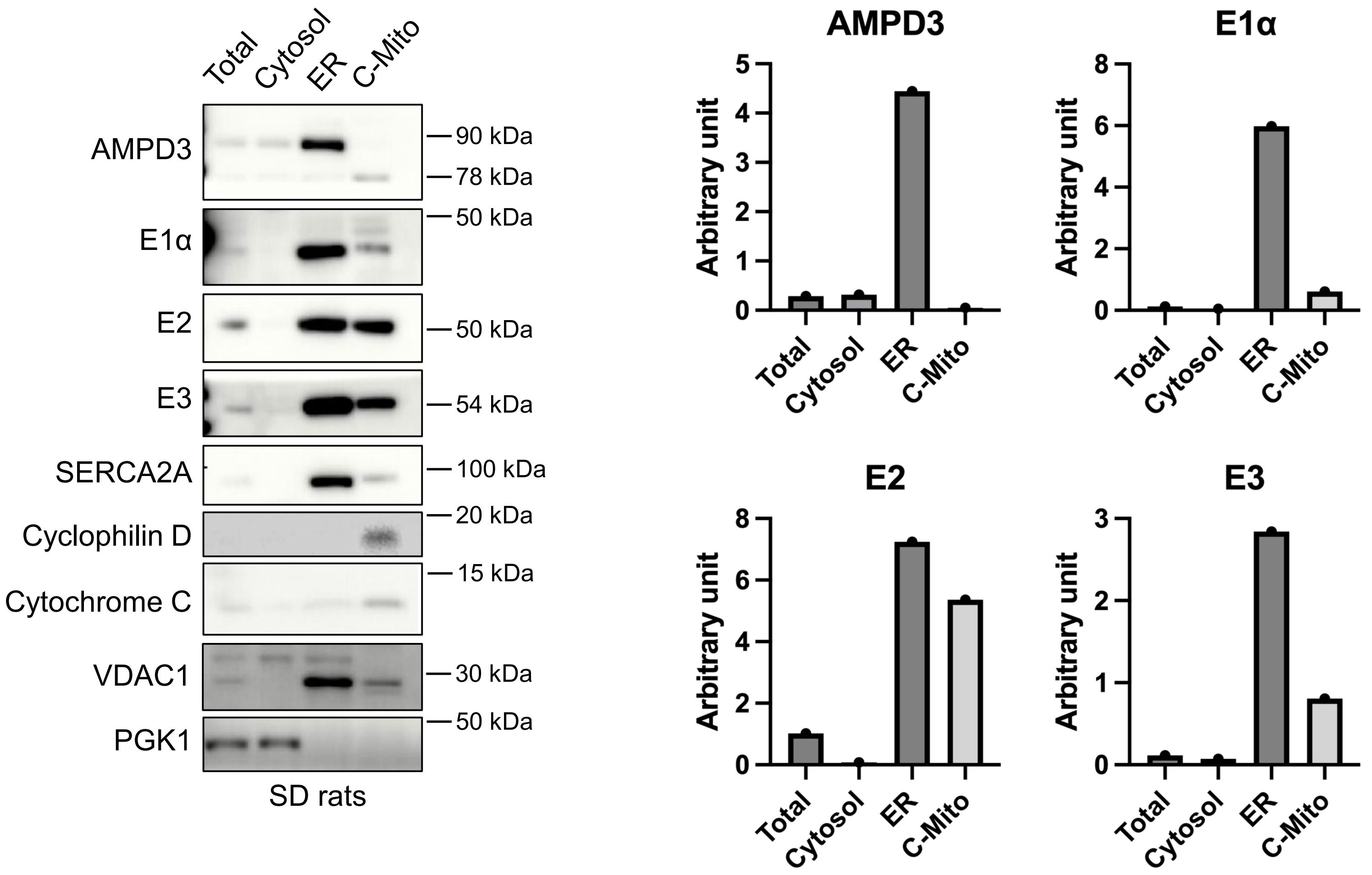

Supplementary Fig. S3. Representative Western blot showing AMPD3 and BCKDH components in subcellular compartments of hearts from Sprague–Dawley rat and summary of the densitometry analysis. The same amount of protein from each fraction was loaded on the electrophoresis.
