# Supplementary material for: Downregulation of extramitochondrial BCKDH and its uncoupling from AMP deaminase in type 2 diabetic OLETF rat hearts
Source: Physiol Rep. 2023 Feb 17;11(4):e15608. doi: 10.14814/phy2.15608 (PMC9938007; doi:10.14814/phy2.15608)
Supplement: Supplementary file 4 — Figure S4. [file PHY2-11-e15608-s001.pdf]

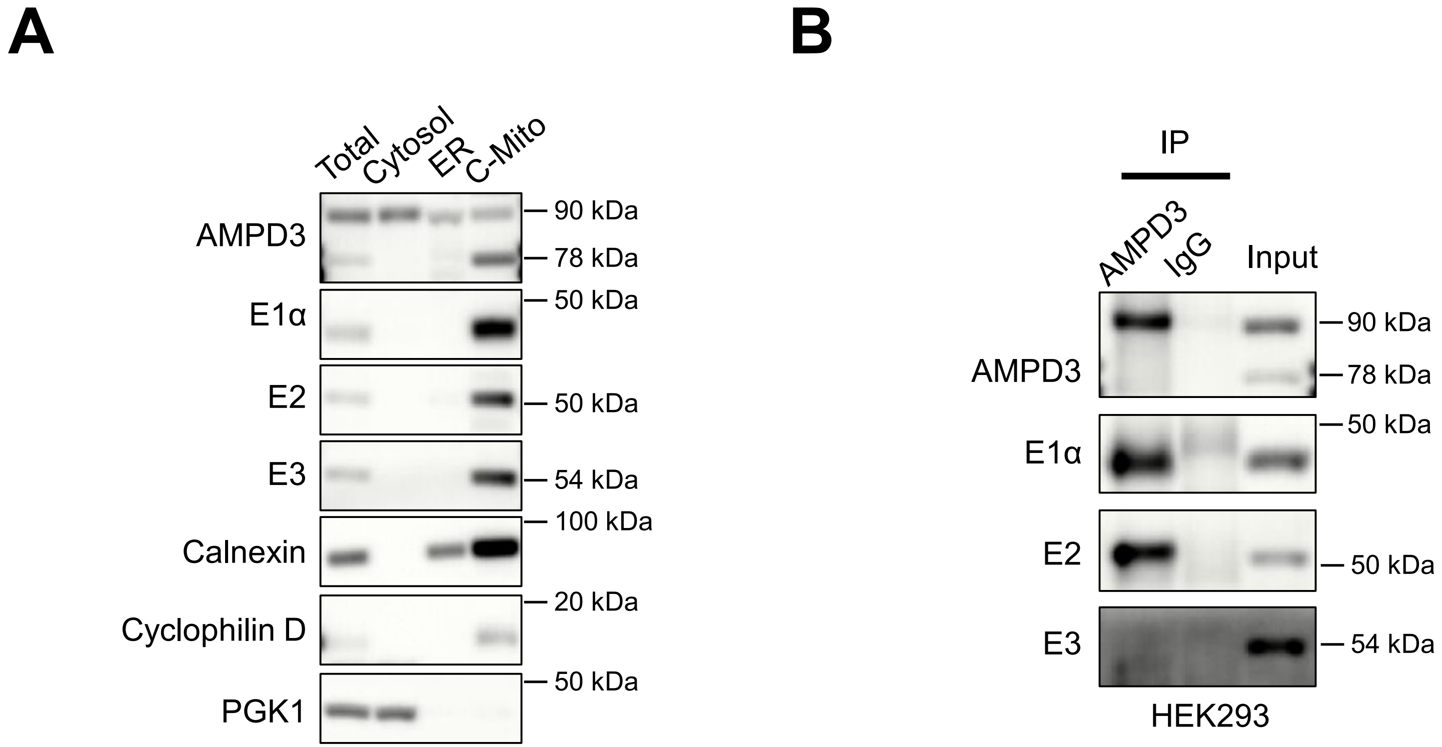

Supplementary Fig. S4. Representative Western blot showing AMPD3 and BCKDH components in subcellular compartments (A) and the AMPD3-BCKDH interaction (B) in HEK293 cells.
