# Supplementary material for: Downregulation of extramitochondrial BCKDH and its uncoupling from AMP deaminase in type 2 diabetic OLETF rat hearts
Source: Physiol Rep. 2023 Feb 17;11(4):e15608. doi: 10.14814/phy2.15608 (PMC9938007; doi:10.14814/phy2.15608)
Supplement: Supplementary file 5 — Figure S5. [file PHY2-11-e15608-s006.pdf]

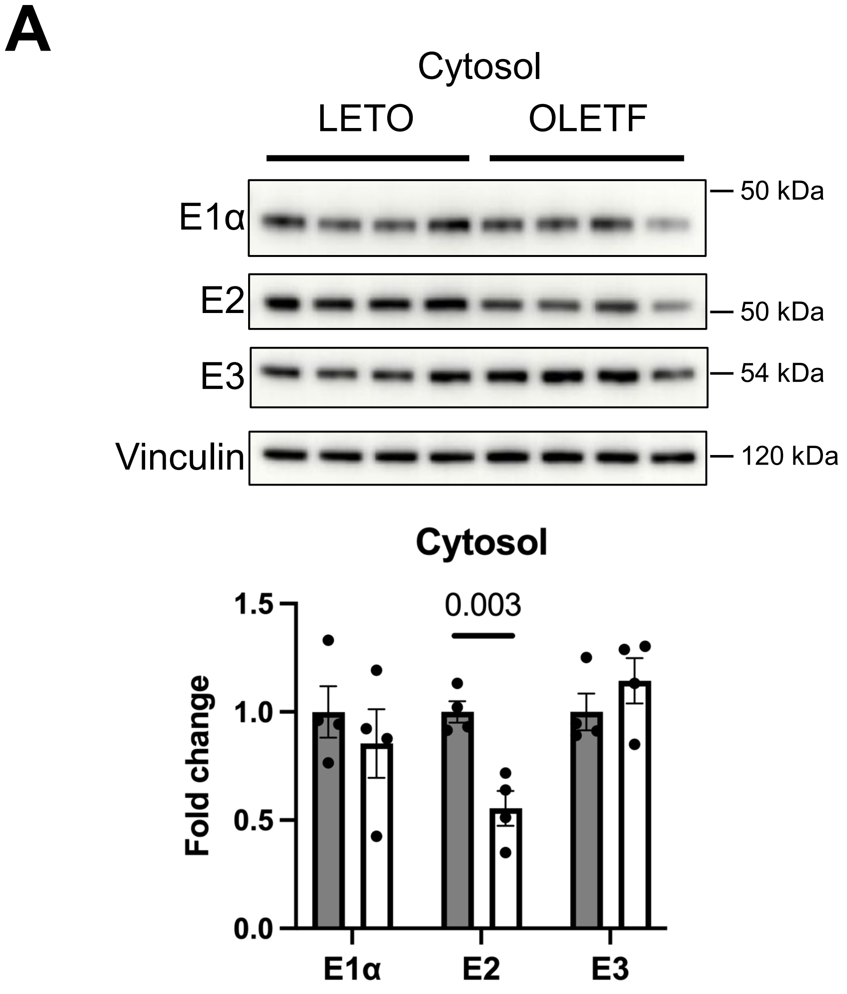

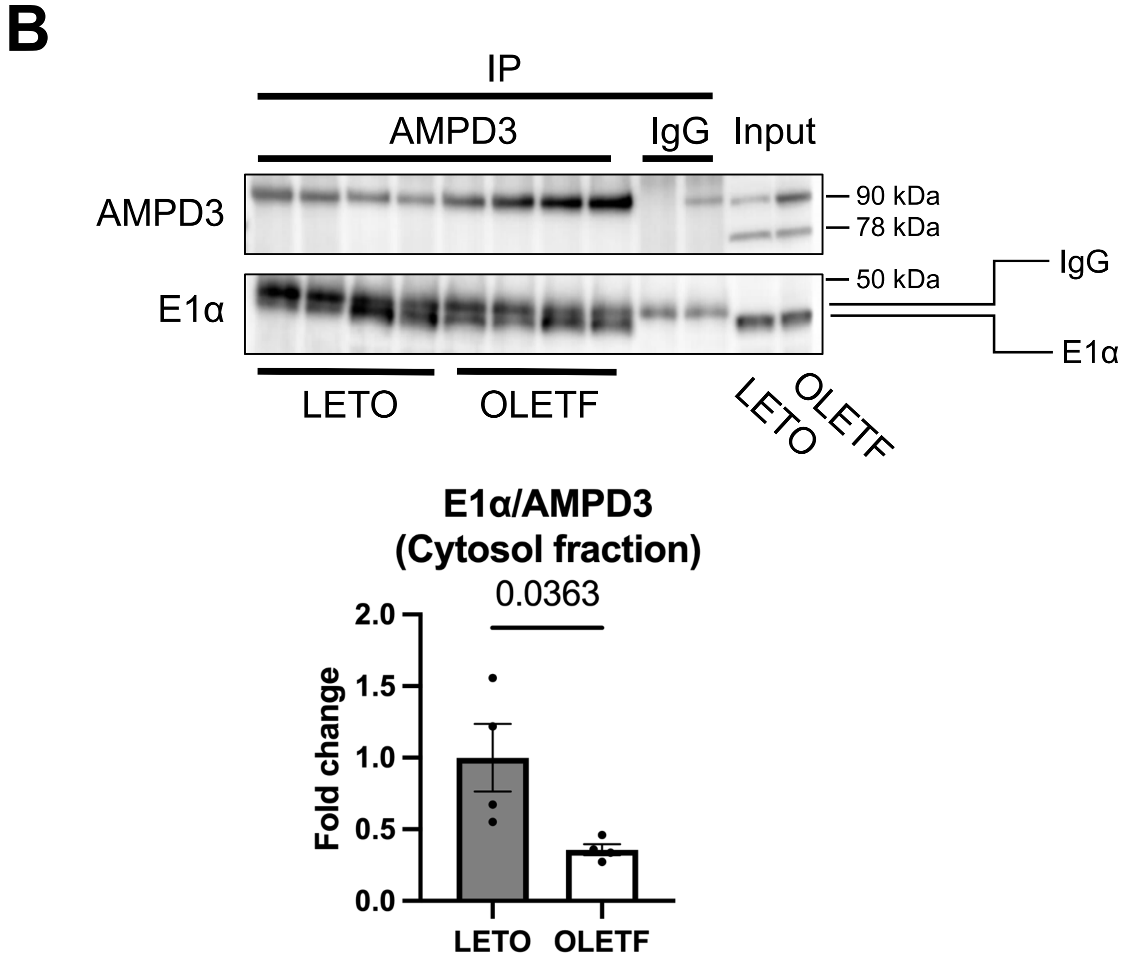

Supplementary Fig. S5. Expression of BCKDH components (A) and the interaction with AMPD3 (B) in the cytosol fraction in LETO and OLETF (N=4 in each group). Data were analyzed by unpaired Student's t test. The p values obtained for comparisons of the groups at both ends of the line are shown.
