# Supplementary material for: Downregulation of extramitochondrial BCKDH and its uncoupling from AMP deaminase in type 2 diabetic OLETF rat hearts
Source: Physiol Rep. 2023 Feb 17;11(4):e15608. doi: 10.14814/phy2.15608 (PMC9938007; doi:10.14814/phy2.15608)
Supplement: Supplementary file 6 — Figure S6. [file PHY2-11-e15608-s008.pdf]

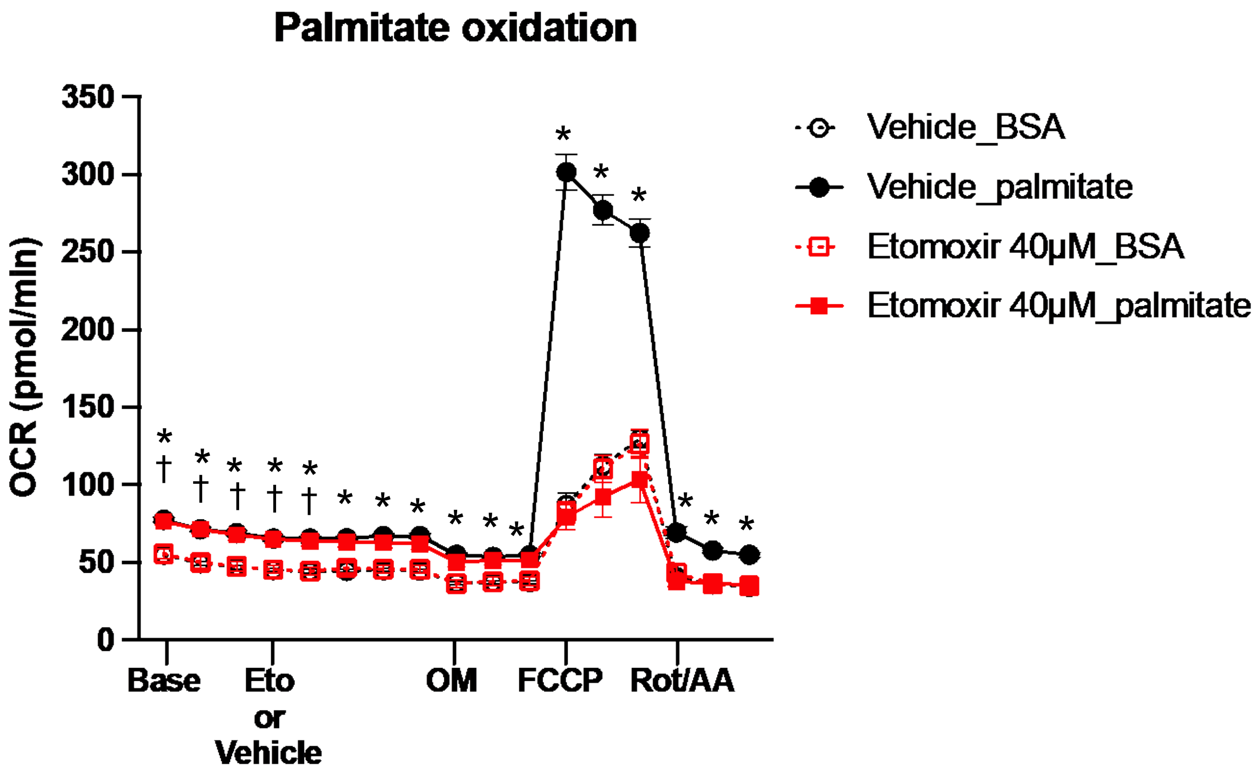

Supplementary Fig. S6. Effect of etomoxir (Eto), a CPT1 inhibitor, on palmitate oxidation assay in NRCMs (N=5-6 in each group). Data were analyzed by two-way repeated-measures ANOVA with Tukey's test for multiple group comparison. *p<0.01 between Vehicle BSA and Vehicle palmitate, †p<0.01 between Etomoxir 40μM BSA and Etomoxir 40μM palmitate.
