# Supplementary material for: Downregulation of extramitochondrial BCKDH and its uncoupling from AMP deaminase in type 2 diabetic OLETF rat hearts
Source: Physiol Rep. 2023 Feb 17;11(4):e15608. doi: 10.14814/phy2.15608 (PMC9938007; doi:10.14814/phy2.15608)
Supplement: Supplementary file 7 — Figure S7. [file PHY2-11-e15608-s002.pdf]

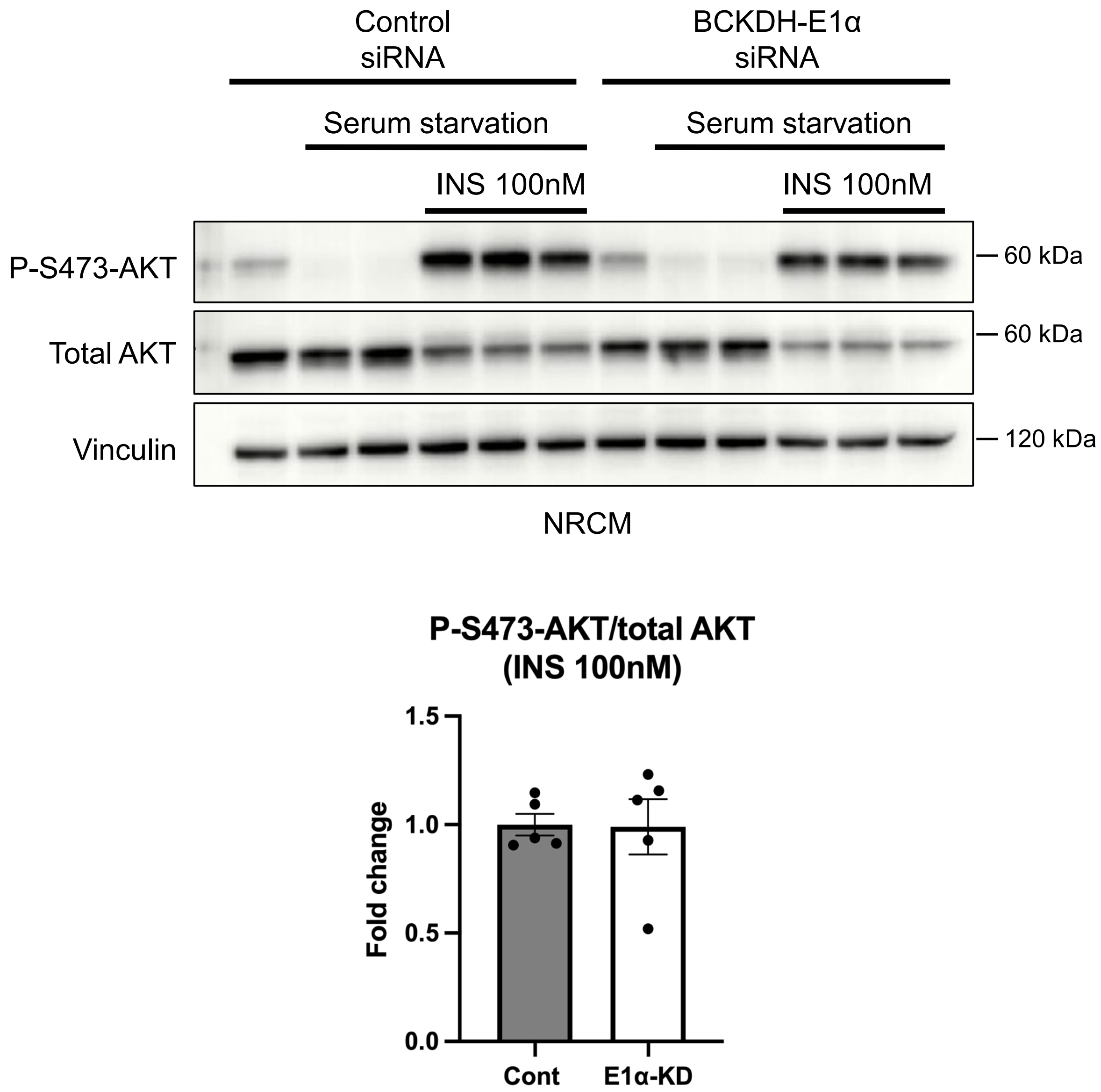

Supplementary Fig. S7. Phosphorylation of AKT in response to insulin in NRCMs with or without BCKDH-E1α knockdown (N=5 in each group). Data were analyzed by unpaired Student’s t test.
