# Supplementary material for: Downregulation of extramitochondrial BCKDH and its uncoupling from AMP deaminase in type 2 diabetic OLETF rat hearts
Source: Physiol Rep. 2023 Feb 17;11(4):e15608. doi: 10.14814/phy2.15608 (PMC9938007; doi:10.14814/phy2.15608)
Supplement: Supplementary file 8 — Figure S8. [file PHY2-11-e15608-s005.pdf]

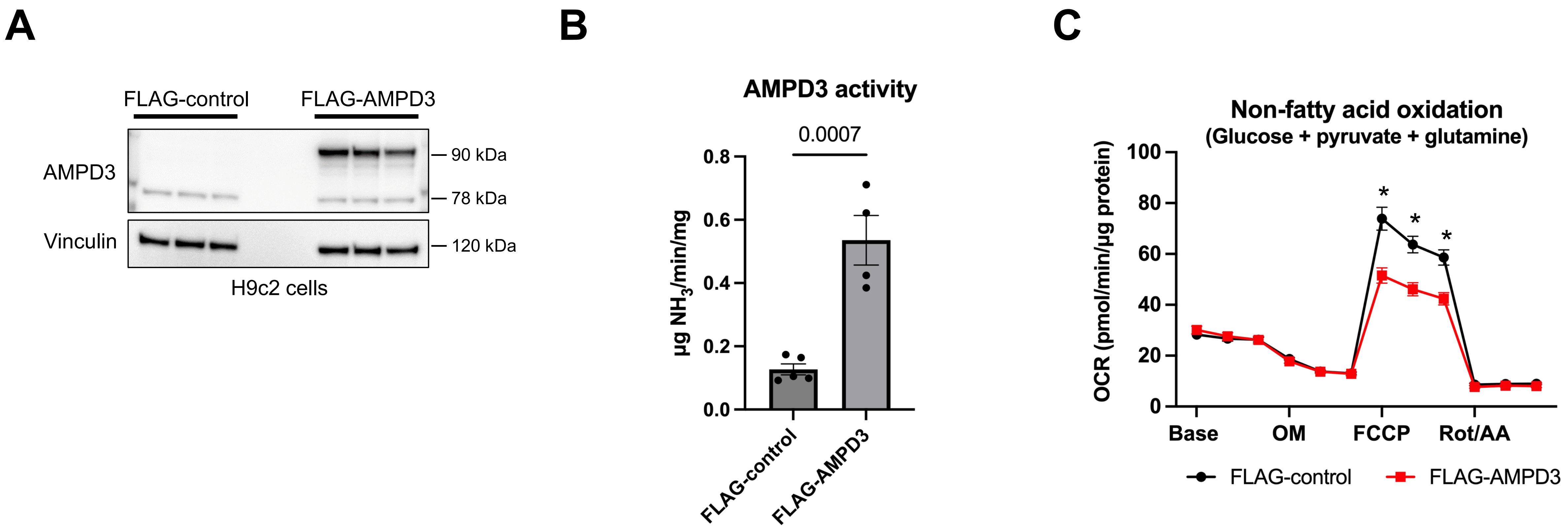

Supplementary Fig. S8. (A-C) Representative Western blot showing AMPD3 (A; N=3 in each group); AMPD3 activity (B; N=4-5 in each group); and oxygen consumption rate (OCR) (C; N=6 in each group. Reproducibility was confirmed in 2 biological replicates) in H9c2 cardiomyoblasts with or without AMPD3 overexpression. Data were analyzed by unpaired Student's t test (B) or two-way repeated-measures ANOVA (C). The p values obtained for comparisons of the groups at both ends of the line are shown. *$p<0.05$ between FLAG-control and FLAG-AMPD3.
