# Supplementary material for: Downregulation of extramitochondrial BCKDH and its uncoupling from AMP deaminase in type 2 diabetic OLETF rat hearts
Source: Physiol Rep. 2023 Feb 17;11(4):e15608. doi: 10.14814/phy2.15608 (PMC9938007; doi:10.14814/phy2.15608)
Supplement: Supplementary file 9 — Table S1. [file PHY2-11-e15608-s007.pdf]

Supplementary Table S1. Proteomic analyses of AMPD3 immunoprecipitates in the LETO and OLETF rat heart lysates.

	Description	Coverage [%]	# Peptides	Score Sequest HT
	LETO			
1	Clathrin heavy chain 1	49	60	327.54
2	[3-methyl-2-oxobutanoate dehydrogenase [lipoamide]] kinase, mitochondrial	61	15	206.11
3	AMP deaminase 3	44	30	160.08
4	Dihydrolipoyl dehydrogenase, mitochondrial	29	12	73.83
	OLETF			
1	Actin, aortic smooth muscle	47	14	129.55
2	AMP deaminase 3	34	24	115
3	Tubulin beta-2A chain	45	15	68.48
4	[3-methyl-2-oxobutanoate dehydrogenase [lipoamide]] kinase, mitochondrial	22	8	42.95

The three candidates showing the greatest interaction with AMPD3 in LETO and OLETF rat hearts were identified by mass spectrometry. Reproducibility was confirmed with N=3 in each group.
